# Global Organization of a Positive-strand RNA Virus Genome

**DOI:** 10.1371/journal.ppat.1003363

**Published:** 2013-05-23

**Authors:** Baodong Wu, Jörg Grigull, Moriam O. Ore, Sylvie Morin, K. Andrew White

**Affiliations:** 1 Department of Biology, York University, Toronto, Ontario, Canada; 2 Department of Mathematics and Statistics, York University, Toronto, Ontario, Canada; 3 Department of Chemistry, York University, Toronto, Ontario, Canada; University of California Riverside, United States of America

## Abstract

The genomes of plus-strand RNA viruses contain many regulatory sequences and structures that direct different viral processes. The traditional view of these RNA elements are as local structures present in non-coding regions. However, this view is changing due to the discovery of regulatory elements in coding regions and functional long-range intra-genomic base pairing interactions. The ∼4.8 kb long RNA genome of the tombusvirus tomato bushy stunt virus (TBSV) contains these types of structural features, including six different functional long-distance interactions. We hypothesized that to achieve these multiple interactions this viral genome must utilize a large-scale organizational strategy and, accordingly, we sought to assess the global conformation of the entire TBSV genome. Atomic force micrographs of the genome indicated a mostly condensed structure composed of interconnected protrusions extending from a central hub. This configuration was consistent with the genomic secondary structure model generated using high-throughput selective 2′-hydroxyl acylation analysed by primer extension (i.e. SHAPE), which predicted different sized RNA domains originating from a central region. Known RNA elements were identified in both domain and inter-domain regions, and novel structural features were predicted and functionally confirmed. Interestingly, only two of the six long-range interactions known to form were present in the structural model. However, for those interactions that did not form, complementary partner sequences were positioned relatively close to each other in the structure, suggesting that the secondary structure level of viral genome structure could provide a basic scaffold for the formation of different long-range interactions. The higher-order structural model for the TBSV RNA genome provides a snapshot of the complex framework that allows multiple functional components to operate in concert within a confined context.

## Author Summary

The genomes of many important pathogenic viruses are made of RNA. These genomes encode viral proteins and contain regulatory sequences and structures. In some viruses, distant regions of the RNA genome can interact with each other via base pairing, which suggests that certain genomes may take on well-defined conformations. This concept was investigated using a tombusvirus RNA genome that contains several long-range RNA interactions. The results of microscopic and biochemical analyses indicated a compact genome conformation with structured regions radiating from a central core. The structural model was compatible with some, but not all, long-range interactions, suggesting that the genome is a dynamic molecule that assumes different conformations. The analysis also revealed new structural features of the genome, some of which were shown to be functionally relevant. This study advances our understanding of the role played by global structure in virus genome function and provides a model to further investigate its in role virus reproduction. We anticipate that organizational principles revealed by this investigation will be applicable to other viruses.

## Introduction

Many viruses possess RNA genomes, and those with single-stranded plus-sense RNA genomes represent an important subgroup that includes many pathogenic plant, animal, and human viruses. These RNA genomes serve the traditional role as the blueprint for viral proteins; however, they also contain cis-acting RNA elements (RE) that direct different viral processes, such as protein translation, genome replication and transcription of subgenomic (sg) mRNAs [1]–[3]. Accordingly, REs need to be structurally and functionally integrated with coding sequences. One solution utilized is to position REs in non-coding regions, thereby physically separating coding and RE functions. Indeed, many viruses use this strategy by situating their REs terminally in 5′- and 3′-untranslated regions (UTRs) or/and internally within inter-cistronic regions [1], [3]. One drawback to this approach is that the size and/or location of the non-coding regions can be limiting. Thus, to extend this range, many plus-strand RNA viruses also position REs within coding regions [1].

One potential drawback of having an RE in a coding region is that it physically couples distinct activities to the same RNA sequence; therefore, some compromise in one or both functions may be required. Additionally, in some cases the relative location of REs within a genome may not be optimal for their activity, thus compensatory measures may be required. One strategy used by many RNA viruses to deal with suboptimally positioned REs is to reorganize their relative location within the genome via intramolecular long-range RNA-RNA interactions [4]. This tactic is employed by a number of different plant viruses to mediate translation of viral proteins from their uncapped and nonpolyadenylated genomes. Specifically, luteoviruses [5], tombusviruses [6], [7], carmoviruses [8] and umbraviruses [9] recruit translation initiation factors to a 3′-proximal RE, termed a 3′ cap-independent translational enhancer (3′CITE), that interacts with the 5′UTR via a long-range RNA-RNA interaction to mediate translation initiation. Interactions between genomic termini have been identified in other types of plus-strand RNA viruses, such as hepatitis C virus (HCV) [10] and picornaviruses [11], but their roles remain to be fully elucidated. Long-range interactions are also used for viral RNA genome replication in bacterial (*i.e.* phage Q-beta) [12], plant (*i.e.* tombusviruses) [13] and animal viruses (*i.e.* flaviviruses) [14], where viral polymerase binding sites are located 5′-terminally or internally in the RNA genome and the bound polymerase is repositioned near the 3′ end of the genome by long-range interactions, thereby allowing for initiation of minus-strand synthesis. In other cases, long-distance RNA-based interactions can themselves form functional RNA structures, as seen in insect nodaviruses [15], animal coronaviruses [16], [17] and plant tombusviruses [18], [19]. For these viruses, the interactions form RNA structures that act as terminators for the viral polymerase during minus-strand synthesis, which then lead to formation of truncated minus-strand templates used to transcribe viral sg mRNAs. Very recently, a second long-range interaction spanning ∼26 kb was identified in transmissible gastroenteritis coronavirus that was required for efficient transcription of its sg mRNA encoding the N gene [20]. As coronaviruses transcribe numerous distinct sg mRNAs, this finding suggests that their genomes may harbour a large number of long-distance interactions. The prevalence of long-range RNA-RNA interactions in a variety of viruses in both coding and non-coding regions suggests that their RNA genomes must maintain a considerable level of global organization. Recently, the secondary structure of the entire human immunodeficiency virus-1 (HIV-1) RNA genome was reported, which revealed many novel structural features within the global genomic context [21]. However, for standard plus-strand RNA viruses (i.e. non-retroviral), no detailed genome-scale models of higher-order structure exist, thus our understanding of genome architecture and dynamics for this important class of virus is very limited.

**Figure 1:**
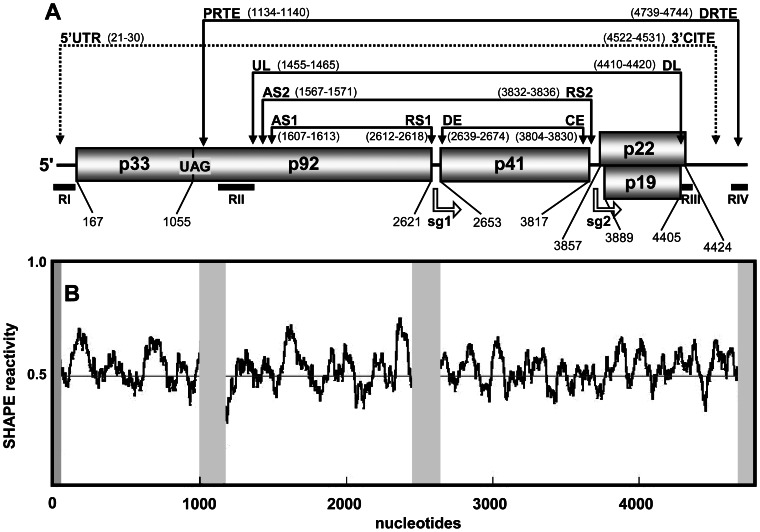
TBSV RNA genome structure. (**A**) Linear representation of the TBSV plus-strand RNA genome. Encoded proteins are shown as boxes with molecular masses (in thousands). The genomic coordinates for the start and stop codons for each encoded protein are shown below the genome. Also shown below are the relative locations of the initiation sites for transcription for sg mRNAs (represented as open arrows and labeled sg1 and sg2), as well as regions (RI through RIV) that are present in prototypical TBSV defective interfering (DI) RNAs (shown as horizontal black bars). The relative locations of intra-genomic long-range RNA-RNA interactions are shown by the labeled double-headed arrows above the genome. Each double-headed arrow connects a complementary cognate sequence pair identified by their acronyms. The coordinates for complementary segments are provided in parentheses. See text for details. (**B**) Median SHAPE reactivities along the TBSV RNA genome based on a 75 nt long moving window. The overall average SHAPE reactivity value was 0.488 and is represented in the graph as a red line. Grey shading indicates regions for which no SHAPE reactivity was recovered.

Tombusviruses, such as tomato bushy stunt virus (TBSV), are plus-strand RNA viruses that encode five proteins in a ∼4.8 kb long genome [22]. p33 and its readthrough product p92, the RNA-dependent RNA polymerase, are both required for genome replication and sg mRNA transcription, and both of these proteins are translated directly from the genome [23]. The more 3′-proximal p41 is the capsid protein that is translated from sg mRNA1, the larger of two subgenomic messages transcribed during infections [23]. The p19 suppressor of gene silencing and p22 movement proteins are both translated from the smaller sg mRNA2 [23]. Analyses of TBSV and other tombusviruses have led to significant understanding of many aspects of the tombusvirus reproductive cycle [23]. Numerous functional cis-acting REs have been mapped within their genomes, and viral and host proteins involved in mediating infections have been characterized [2], [24]. A particularly intriguing aspect of tombusviruses is that they use a vast network of intra-genomic long-range RNA-RNA interactions to mediate a number of different processes [4]. In total, six different functional long-distance base pairing interactions, each spanning sequences ≥1 kb, have been identified (Figure 1A). Translation of p33 requires the longest interaction, which occurs between the 3′CITE in the 3′UTR and the genomic 5′UTR [25]. Translational readthrough of the p33 stop codon to produce p92 involves an interaction between the proximal readthrough element (PRTE) located 3′ to the stop codon and the distal readthrough element (DRTE) in the 3′UTR [26]. Genome replication requires an interaction between the upstream linker (UL) sequence just 3′ to the essential replication element RII(+)SL and the downstream linker (DL) in the 3′UTR [13]. This latter interaction is also needed for efficient translational readthrough that generates p92 [26]. Transcription of the sg mRNAs utilizes three different interactions; the activator sequence 1-receptor sequence 1 (AS1-RS1) interaction mediates sg mRNA1 transcription [18] and the AS2-RS2 and distal element-core element (DE-CE) interactions facilitate sg mRNA2 transcription [19], [27].

Interestingly, the latter five interactions described are all part of a set of nested interactions, suggesting possible structural order within this grouping (Figure 1A). Additionally, some of the interacting sequences are located relatively close to each other (*i.e.* UL, AS2 and AS1), hinting at possible shared organizational strategies and/or functional or regulatory crosstalk (Figure 1A). Accordingly, we were interested in investigating the genome architecture of a tombusvirus, namely TBSV, in order to gain a better understanding of how local REs and multiple long-range interactions are accommodated and functionally integrated within the genome. The results from our structural analyses indicate that the TBSV genome forms a relatively compact structure that is organized into a number of differently sized domains. The largest domains mediate functional long-range interactions, whereas some of the smaller domains correspond to local REs. The analyses also revealed novel long-range interactions and local structures, some of which were shown to be functional. Collectively, this study provides considerable new insight into the structure and dynamics of a plus-strand RNA genome and supports the intriguing concept that global organization represents an integral component of genome function.

## Results/Discussion

### Microscopic analysis of the TBSV genome

**Figure 2:**
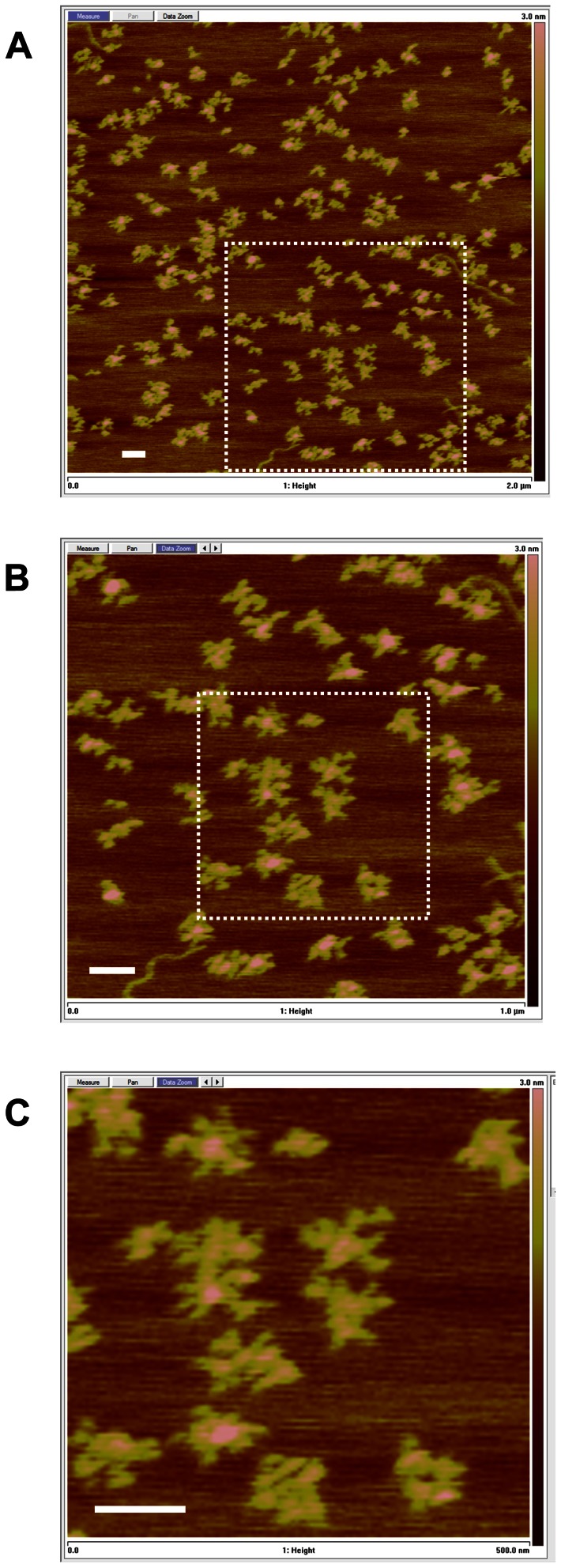
AFM analysis of TBSV RNA genome transcripts. Successive two-fold magnifications of the image in (**A**) are presented in (**B**) and (**C**), with the regions magnified defined by the dotted lines. All scale bars are 100 nm and the Z scale ranges from 0 to 3 nm.

As an initial approach to gain insights into the global organization of the TBSV genome we used atomic force microscopy (AFM). This type of analysis provides information on the overall topography of an RNA molecule adsorbed to a mica surface and has been used successfully to assess global conformations of different viral RNA genomes [28], [29]. Our results revealed that TBSV genomes exist primarily as compact irregular structures, with average maximum diameters of 98 ±12 nm (n = 40) (Figure 2). A theoretical maximum ladder distance (MLD) [30] of 248 bp was calculated for the TBSV genome, which represents the number of base pairs passed when spanning the two furthest points in the optimal structure, predicted by the *RNAstructure* software [31]. With the average rise per base pair in dsRNA corresponding to ∼0.27 nm [32], this MLD converts to an average measurement of ∼67 nm. The MLD average over 1000 suboptimal secondary structures was calculated as 252±6 bp or ∼68 nm. These somewhat lower values are generally consistent with the measured value from AFM, because they do not include increases in length contributed by the unstructured regions connecting the base paired segments. Overall, the estimated dimensions from the *RNAstructure*-predicted configuration and the AFM analysis suggest a compact secondary structure for the TBSV genome.

Due to conformational flexibility, variable surface adsorption, and limits of resolution, a single well-defined structure for all genome molecules was not anticipated. However, what was evident from the AFM analysis was the general conservation of a global organization consisting of irregular extensions emanating from a central core (Figure 2). Some variation from this overall arrangement was evident, but the level of compactness and the presence of multiple tethered substructures were typically maintained. These results suggest that the TBSV genome assumes a mostly condensed structure that is composed of interconnected domain-like protrusions extending from a central hub. In this respect, its genome structure is most similar to the more compact structures observed via AFM for HCV and Hepatitis G virus RNA genomes, and contrasts the more extended arrangements seen for poliovirus and rubella virus RNA genomes [28], [29]. Extended genome configurations suggest smaller consecutive locally-folded domains, whereas more condensed structures are consistent with genome configurations that include multiple long-distance RNA-RNA interactions [28]. The compact structures observed for the TBSV genome are in agreement with the latter concept and are consistent with the numerous long-range interactions known to occur in this genome [4].

### SHAPE analysis of the TBSV genome

We next sought to determine the RNA secondary structure of the TBSV genome using high-throughput selective 2′-hydroxyl acylation analysed by primer extension (SHAPE) [33]. Full-length transcripts of the TBSV genome were denatured, snap-cooled, and then refolded in an effort to (i) minimize intermolecular interactions, (ii) release misfolded molecules from kinetic traps, and (iii) promote formation of the most stable secondary structure(s). The latter event would presumably reduce structural diversity in the population and allow for more readily interpretable results. As transcripts of plus-strand RNA virus genomes are infectious when transfected into cells, the refolded viral RNA was tested for replication in plant protoplasts. Progeny genomes from refolded or untreated transcripts accumulated to equivalent levels (Figure S1), indicating that the folding step did not interfere with virus infectivity. Refolded RNA transcripts were treated with 1-methyl-7-nitroisatoic anhydride, which preferentially acylates the 2′-OH position of riboses associated with conformationally flexible nucleotides [33]. Accordingly, heavily modified positions are predicted to correspond to nucleotides that are primarily single-stranded, while unmodified or weakly modified residues are considered to be mainly paired. Inflexible regions can also correspond to non-Watson/Crick base pairs or tertiary interactions. Modified positions in the viral genome were detected and quantified using primer extension and capillary electrophoresis [33].

**Figure 3:**
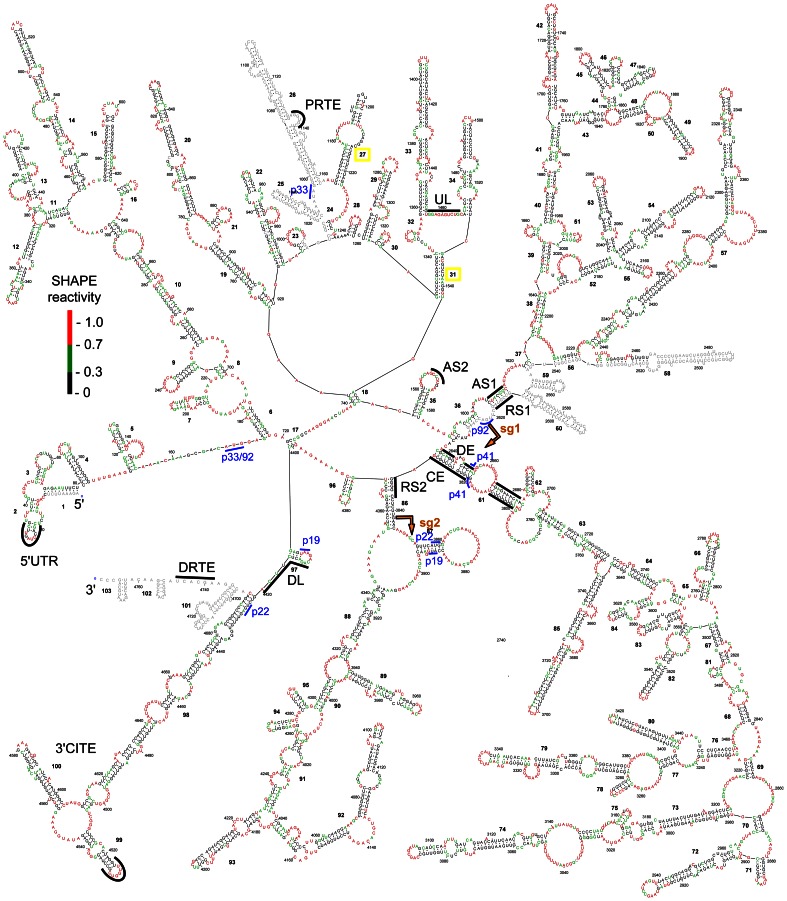
SHAPE-guided secondary structure model for the TBSV RNA genome. Nucleotides are colour-coded according to their SHAPE reactivities as indicated in the colour key. Nucleotides in grey correspond to regions for which no SHAPE data were available. Nucleotide coordinates and base paired (*i.e.* stem or helical) regions are numbered in regular and bold type, respectively. The complementary segments known to form functional long-range RNA-RNA interactions are labeled and delineated by black lines. Start and stop codons for viral ORFs are labeled and defined by blue bars, while initiation sites for sg mRNA transcription are denoted by gold arrows. See text for details.

SHAPE reactivity data were collected for ∼91% of the 4778 nt long TBSV genome, with the final values derived from the averages from two separate SHAPE analyses (Table S1). Figure 1B shows median SHAPE reactivities along the TBSV RNA genome based on a 75 nt long moving window. The average SHAPE reactivity based on this analysis was 0.488 (Figure 1B). Two regions corresponding to positions 1008–1163 and 2464–2632 could not be assessed due to strong stops in the reverse transcription reactions (Figure 1B). Also, no reactivity data were collected for the 5′-terminal 9 nt and the 3′-terminal 83 nt of the genome; the latter was due to the lack of a priming site available downstream of this segment. The absence of the terminal information was not problematic because prior analyses had thoroughly defined the functionally relevant secondary and tertiary structures in the 5′UTR and 3′UTR [34]–[36]. For the internal segments where no SHAPE data were available, structure prediction was guided by thermodynamics-based parameters only. The SHAPE reactivity data collected for the rest of the genome were normalized and incorporated into the thermodynamics-based *RNAstructure* software using a pseudo free-energy change term [33]. Subsequently, this program, guided by both thermodynamics and SHAPE data, was used to predict the RNA secondary structure for the TBSV genome. No limit was placed on the maximum base pairing distance for the folding analysis, because many experimentally verified long-range interactions are known to form in the TBSV genome [4]. To prevent improbable long-range interactions between internal regions and experimentally-verified local structures situated at either terminus of the genome, formation of the confirmed local RNA structures in the 5′UTR (nts 1–166) and 3′UTR (nts 4697–4778) was added as a constraint in the folding analysis. This restriction was introduced because prior comprehensive mutational analyses of these terminal structures indicated that it is highly improbable that base paired regions within these local structures participate in alternative functionally relevant long-range base pairing interactions [34]–[38]. Based on the parameters described above, an RNA secondary structure model for the entire TBSV genome was predicted by SHAPE-guided *RNAstructure*-based analysis (Figure 3). It should be noted that the RNA structure generated included only secondary structures formed by canonical base pairs, as this algorithm does not predict non-canonical interactions or tertiary structures such as pseudoknots.

### Secondary structure model for the TBSV RNA genome

Initially, we were interested in determining whether the SHAPE data contributed significantly to the refinement of the genomic structural model. The model that included the SHAPE data (*i.e.* SHAPE-plus: SHAPE and thermodynamics) predicted 60.4% of the genome to be base paired, while exclusion of the SHAPE data (*i.e.* SHAPE-minus: thermodynamics-only) yielded a structure with 65.5% base pairing. Accordingly, addition of the SHAPE data reduced overall predicted pairing by ∼5%. Approximately 70% of the base pairs in the SHAPE-plus structure were identical to those in the SHAPE-minus structure; therefore inclusion of the SHAPE data did result in altered pairing schemes and model refinement. Interestingly, this latter value is higher than the corresponding 47% determined during structural analysis of the HIV-1 genome [21]. Thus, compared to HIV-1, the SHAPE data for the TBSV genome were more consistent with the thermodynamics-only prediction.

At a global level, the structural model revealed distinct sub-regions of RNA secondary structure that we herein define as RNA domains. The boundaries (*i.e.* closing stems) of several of these domains were closely associated, leading to their emergence from a common area and an overall floret-like organization (Figure 3). Notably, this arrangement is consistent with the structures observed by AFM indicating domain-like protrusions emanating from a central core (Figure 2). The MLD calculated for the SHAPE-directed optimal and 1000 suboptimal structures was 216±19 bp (∼58 nm), consistent with compact structures. Such compressed arrangements differ from the more extended beads-on-a-string-like genomic organization proposed for HIV based on high-throughput SHAPE analysis, implying that TBSV and HIV have rather different genomic organizations [21]. Also, a very recent report of the in vitro structure of the ∼1 kb long plus-strand RNA genome of satellite tobacco mosaic virus (unrelated to tombusviruses) predicted a highly extended secondary structure [39] that contrasts the highly condensed structure proposed for TBSV.

**Figure 4:**
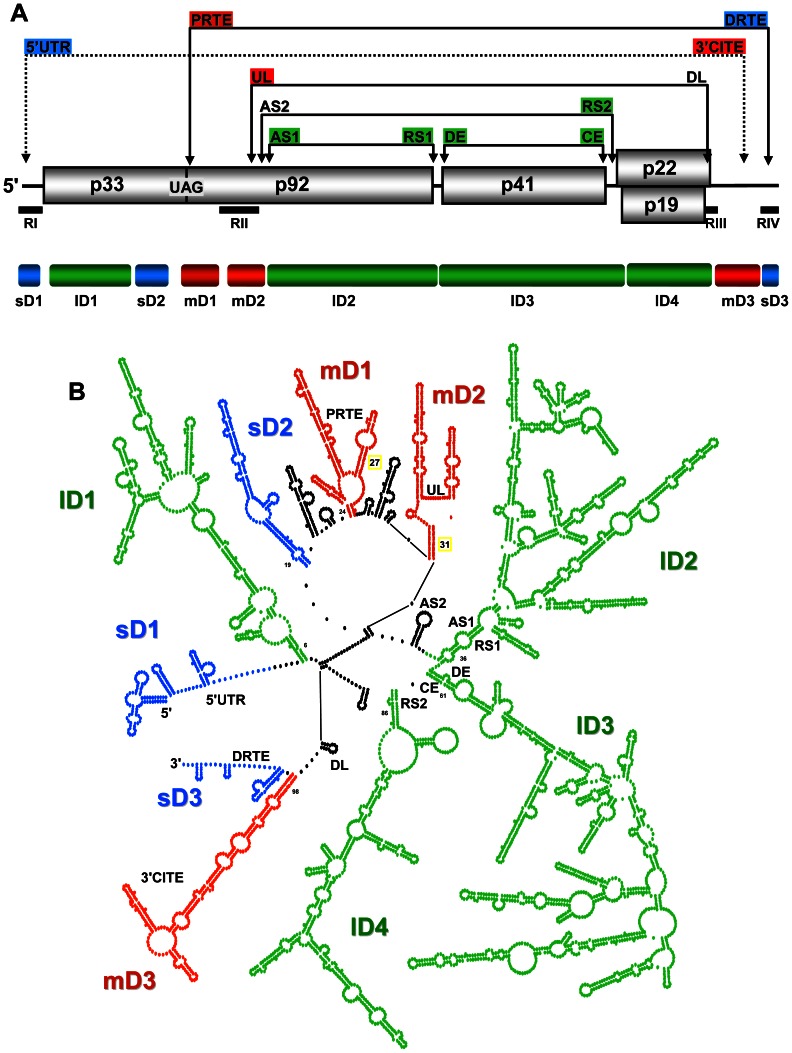
Proposed RNA domains in the TBSV genome. (**A**) Linear representation of the TBSV RNA genome. The genomic regions corresponding to defined domains are labeled and represented by colour coded bars below the genome (blue, small; red, medium; green, large). Above the genome, the partner sequences in long-range interactions are colour-coded according to the domain in which they reside. (**B**) Simplified version of the TBSV genome secondary structure. Each dot represents a nucleotide and domains are labeled and colour-coded as in panel (A). Inter-domain regions are depicted in black. The regions containing complementary segments known to form functional long-range RNA-RNA interactions are labeled.

In TBSV, the RNA domains could be divided into groups based on their relative sizes (*i.e.* small, 80–200 nt; medium, 200–500 nt; large, 500–2000 nt). Accordingly, three small, three medium, and four large domains were defined (Figure 4). The three small domains (sD) corresponded to the 5′UTR (termed sD1, 166 nt long, genome coordinates 1–166), the terminal 81 nts of the 3′-UTR (sD3, 81 nt, 4697–4777), and an internal region in the p33 ORF (sD2, 174 nt, 747–921) (Figure 4). The terminal sD1 and sD3 were functionally characterized previously and are involved in genome replication (sD1 and sD3) [34]–[38], translation (sD1) [25], [38] and readthrough (sD3) [26], whereas the role of sD2 is currently unknown. The three medium domains (mD) were derived from a region in the p92 ORF that overlapped with the p33 ORF stop codon (mD1, 229 nt, 1013–1241), a region downstream of the p33 stop codon in the p92 ORF (mD2, 216 nt, 1328–1543), and the 5′-portion of the 3′UTR (mD3, 275 nt, 4421–4695) (Figure 4). mD1 contains a prominent stem-loop structure containing the PRTE that interacts with the DRTE in SD3 to mediate readthrough [26] (Figure 4). mD2 contains the large stem-loop structure RII(+)-SL and the UL sequence, both of which are involved in genome replication [13], [40], [41]. mD3 corresponds to the 3′CITE that is important for translational enhancement [25], [38]. Lastly, the four large domains (lD) correspond to the 5′-proximal two-thirds of the p33 ORF (lD1, 547 nt, 172–718), the 3′-proximal two-thirds of the readthrough portion of the p92 ORF (lD2, 1047 nt, 1589–2635), the CP ORF (lD3, 1192 nt, 2639–3830), and the overlapping p22/19 ORFs (lD4, 540 nt, 3832–4371) (Figure 3 and 4). Base paired AS1 and RS1 elements, which mediate sg mRNA1 transcription [18], were present at the ends of lD2. Similarly, base paired DE and CE segments, which facilitate sg mRNA2 transcription [19], [27], closed the lD3 domain. At the 5′ end of the 1D4 domain, the RS2 sequence involved in sg mRNA2 transcription was paired to the 3′-end of 1D3, instead of to its far-upstream partner sequence AS2, suggesting that cognate pairs may have alternative partner sequences (Figure 3 and 4). The internal regions of the three consecutive lDs do not contain any known functional RNA elements; thus, their primary function may be to assist in the global positioning of transcriptional elements, as is discussed in more detail in the subsequent section.

The linear 5′-to-3′ order of the ten defined domains is sD1-lD1-sD2-mD1-mD2-lD2-lD3-lD4-mD3-sD3, with inter-domain lengths ranging from as little as 1 nt (between lD3 and lD4, and between mD3 and sD3) up to 91 nts (between sD2 and mD1). Analysis of the predicted structures of the optimal and 1000 suboptimal SHAPE-plus structures revealed that the assigned domains, with the exception of mD1, were well maintained in the population (Figure S2). Some of the inter-domain regions contained RNA elements known to be functional (Figure 4). For example, the inter-domain region between mD2 and lD2 contains the transcriptional AS2 element that is present in the loop of a local stem-loop RNA structure that can pair with its downstream partner sequence RS2 [19] (Figure 4). However, in the structure, the RS2 sequence is paired with a non-cognate partner sequence at the base of lD4 (Figure 3). The inter-domain region between lD4 and mD3 also contains a functional element, the DL sequence, which is complementary to the UL sequence in mD2 (Figure 4). This interaction is required for genome replication [13] as well as translational readthrough [26]. However, in the structure, some of DL sequence is part of a small local stem-loop structure (Figure 4). Collectively, examination of the structural model for the TBSV genome revealed several interesting features: (i) three of the four lDs are grouped consecutively, are closely linked (*i.e.* have small connecting inter-domain segments), and are positioned more centrally in the genome; (ii) ∼66% of the genome is present in the four lDs, internal regions of these domains do not contain known functional RNA elements, and each domain corresponds to a different viral ORF; (iii) the sDs and mDs tend to be located closer to genomic termini; (iv) known functional RNA elements are located in both domain and inter-domain regions; (v) long-range RNA-RNA interactions are evident throughout the structure, but only a third of the known functional long-distance RNA-RNA interactions (i.e. AS2-RS2 and DE-CE) are present in the model.

### Genomic context of RNA elements involved in known long-range RNA-RNA interactions

Previous studies uncovered six different functional long-range RNA-RNA interactions that occur during the TBSV reproductive cycle (Figure 4A). Interestingly, four of these six interactions were not predicted in our structural model, namely, 5′UTR-3′CITE, UL-DL, PRTE-DRTE, and AS2-RS2 interactions (Figure 4B). It is possible that these interactions did not form under the conditions tested or, alternatively, they did form, but limitations in the algorithm prevented their incorporation into the model. Indeed, all of the missing interactions could involve tertiary structures that are not predicted by the program. To try to distinguish between these two possibilities, the shape reactivities of the partner sequences in these interactions were assessed under the premise that high SHAPE reactivities for one or both partner sequences would be consistent with the absence of the cognate interaction, regardless of predictive limitations in the algorithm. Relative reactivities were not available for the PRTE and DRTE due to “blind spots” in the analysis, as explained earlier. Of the remaining partner elements, high reactivities (*i.e.* ≥0.7) were abundant in both complementary sequences for the 5′UTR-3′CITE interaction, the UL sequence, and the AS2 element (Table S2), consistent with their predicted unpaired status in the structure (Figure 3 and 4B). In contrast, DL and RS2 (the partner sequences for UL and AS2, respectively) exhibited relatively lower reactivity scores in general (Table S2) and, consequently, were predicted to form a local hairpin structure or to pair with non-cognate partner sequences, respectively (Figure 3 and 4B). Overall, the relative reactivities of one or both of the partner sequences in the 5′UTR-3′CITE, UL-DL, and AS2-RS2 interactions are consistent with both the lack of their formation in the structure model and the presence of non-cognate pairing. Furthermore, analysis of the 1000 suboptimal SHAPE-plus structures did not predict formation of any of these interactions (Figure S3). Indeed, it may not be possible for all six distinct interactions to occur simultaneously, due to local and/or global conformational constraints. Moreover, mutual exclusion between certain interactions could be advantageous for coordinating sequential or opposing processes. Interestingly, the UL-DL interaction was predicted to occur in the SHAPE-minus structure (Figure S4), indicating that thermodynamics-only structural prediction can be helpful for identifying alternative conformations that are be biologically relevant. For the SHAPE-plus structure, it is interesting to note that although several of the long-range interactions were not predicted, the cognate partner sequences tended to be in proximity of each other in the predicted secondary structure (Figure 3 and 4). Specifically, the ladder distances separating the unpaired partner sequences of 5′UTR-3′CITE, PRTE-DRTE, UL-DL and AS2-RS2 were, respectively, 78 bp (∼21 nm), 29 bp (∼8 nm), 20 bp (∼5.5 nm) and 9 bp (∼2.5 nm). Such proximity could facilitate the structural transitions required to allow these other interactions to form and suggests that the secondary structure level of viral genome structure could provide a basic scaffold for the formation of different long-range interactions. In this respect, the three consecutive large domains would play major roles in bringing together the majority of the interacting partner sequences (Figure 3 and 4).

The two long-range interactions that were predicted to occur in the structural model correspond to AS1-RS1 and DE-CE (Figure 3 and 4). Interestingly, both of these interactions act to close large domains, lD2 and lD3, respectively, and both are involved in sg mRNA transcription, albeit for different sg mRNAs (*i.e.* sg mRNA1 and 2, respectively). Accordingly, the simultaneous presence of these two interactions may provide a glimpse at what a transcriptionally-active genome looks like. Although the AS2-RS2 interaction is not engaged in this model, the two sequences are juxtaposed by the DE-CE and AS1-RS1 interactions, which could facilitate transient AS2-RS2 pairing. Indeed, the DE-CE interaction is critical for AS2-RS2-dependent sg mRNAs transcription [27] and the current structural model supports the previous hypothesis that the DE-CE interaction functions to position AS2 and RS2 close together within the genome structure [19]. It should be noted that long-range RNA-RNA interactions involved in regulating sg mRNA transcription are not restricted to tombusviruses. Other plus-strand viruses also require such distal intra-genomic interactions for transcription of their sg mRNAs. Interestingly, the corresponding predicted RNA secondary structures in the genomes of the insect nodavirus Flock House virus [15] and the animal coronavirus transmissible gastroenteritis virus [17] revealed a similar general arrangement for the distant interacting sequences as seen for AS1-RS1 and DE-CE in the TBSV genome model. Specifically, the interacting transcriptional segments were located near the closing ends of RNA domains, suggesting that domain formation which sequesters intervening sequences could represents a common conformational strategy used by different RNA viruses for uniting distal regulatory RNA sequences.

### Insights into formation of a long-range RNA-RNA interaction

**Figure 5:**
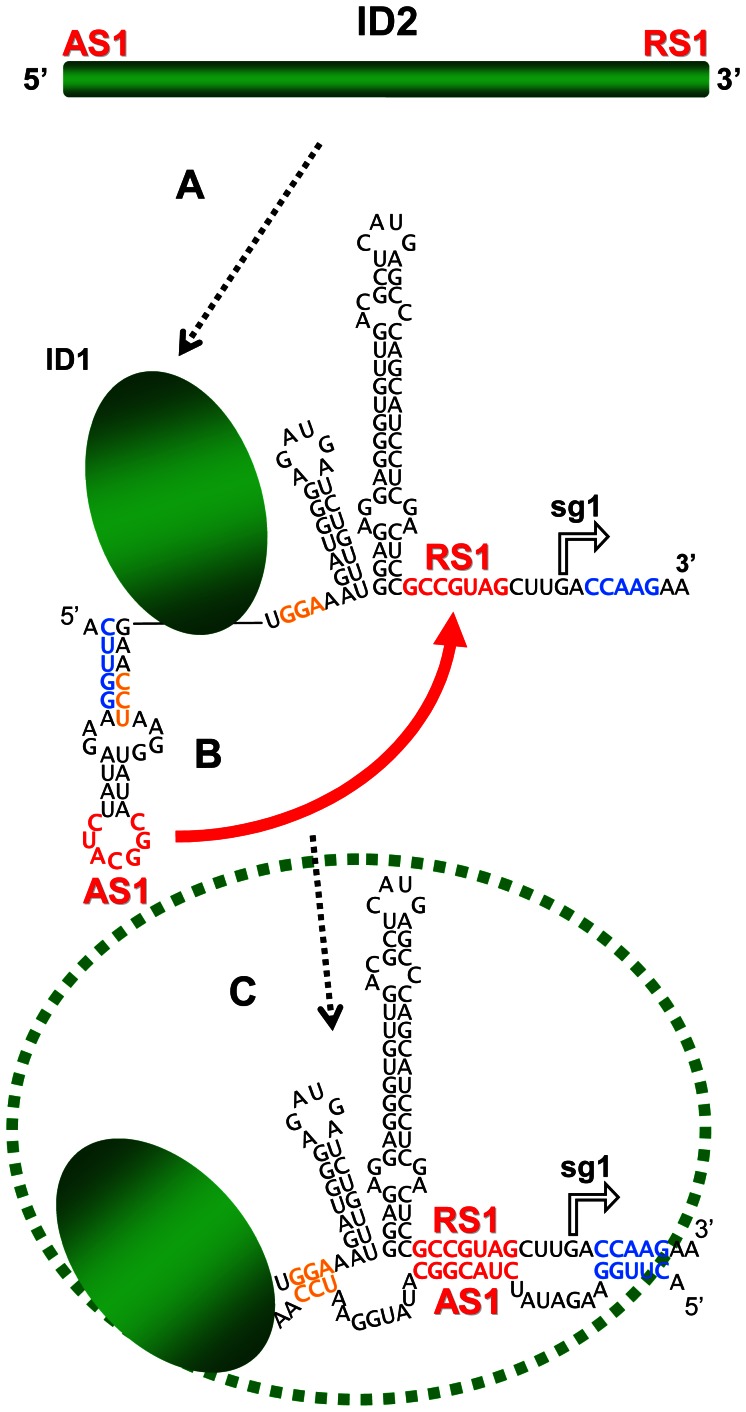
Hypothetical model for formation of AS1-RS1 intra-genomic long-range RNA-RNA interaction. (**A**) Linear representation of lD2. (**B**) Folding of the internal region of lD2 brings AS1 and RS1 (red nucleotides) in proximity and base pairing between these sequences initiates the interaction (red arrow). (**C**) Melting of the AS1 stem permits it to pair with sequences flanking RS1 (blue and gold nucleotides), thereby allowing for completion of domain formation (as depicted by the green dotted oval).

The AS1-RS1 interaction involving the terminal regions of the large domain lD2 is particularly intriguing. When the AS1 element is considered in its local context (*i.e.* within its directly flanking sequence), it is predicted to be positioned in the loop of a local stem-loop structure (Figure 5A), the formation of which was shown to facilitate sg mRNA1 transcription [42]. Consequently, it was proposed that this stem-loop structure helps to present AS1 in a single-stranded form that promotes its pairing with RS1 [42]. However, in the genomic model, the local AS1-containing stem-loop structure is not predicted and, instead, the sequences flanking AS1 are paired with segments upstream and downstream from RS1 (Figure 4). Accordingly, a sequential pairing pathway can be proposed based on (i) the relative locations of AS1 and RS1 within lD2, (ii) the requirement for the local AS1-containing stem-loop structure for efficient transcription and (iii) the local context of the AS1-RS1 interaction in the structural model. To begin with, the formation of lD2 would act to bring together distally positioned AS1 and RS1 elements (Figure 5A and 5B). The AS1 in the loop of the local stem-loop structure would initiate the interaction by pairing with RS1 (Figure 5B) and, subsequently, the stem would melt and allow those residues to pair with sequences flanking RS1, thereby stabilizing the interaction and forming the closing stems of lD2 (Figure 5C). This pairing scheme is reminiscent of the classical regulatory interaction that takes place in *E. coli* between Hok mRNA and Sok-antisense-RNA, where the initial contact between the loop in a stem-loop within the mRNA and an unpaired region in the antisense RNA is followed by melting of the stem and formation of additional pairing between the two RNAs [43]. However, in this latter case the interaction is intermolecular, and thus dependent on the concentrations of the two RNAs. In contrast, the AS1-RS1 interaction is intramolecular [18]; therefore, its formation would be heavily influenced by genomic context. In this respect, the scenario in Figure 5 provides an example of how both local and large-scale structures could work collectively to mediate structural rearrangements that lead to a functional long-range RNA-RNA interaction.

### Functional relevance of components in the TBSV genome structural model

**Figure 6:**
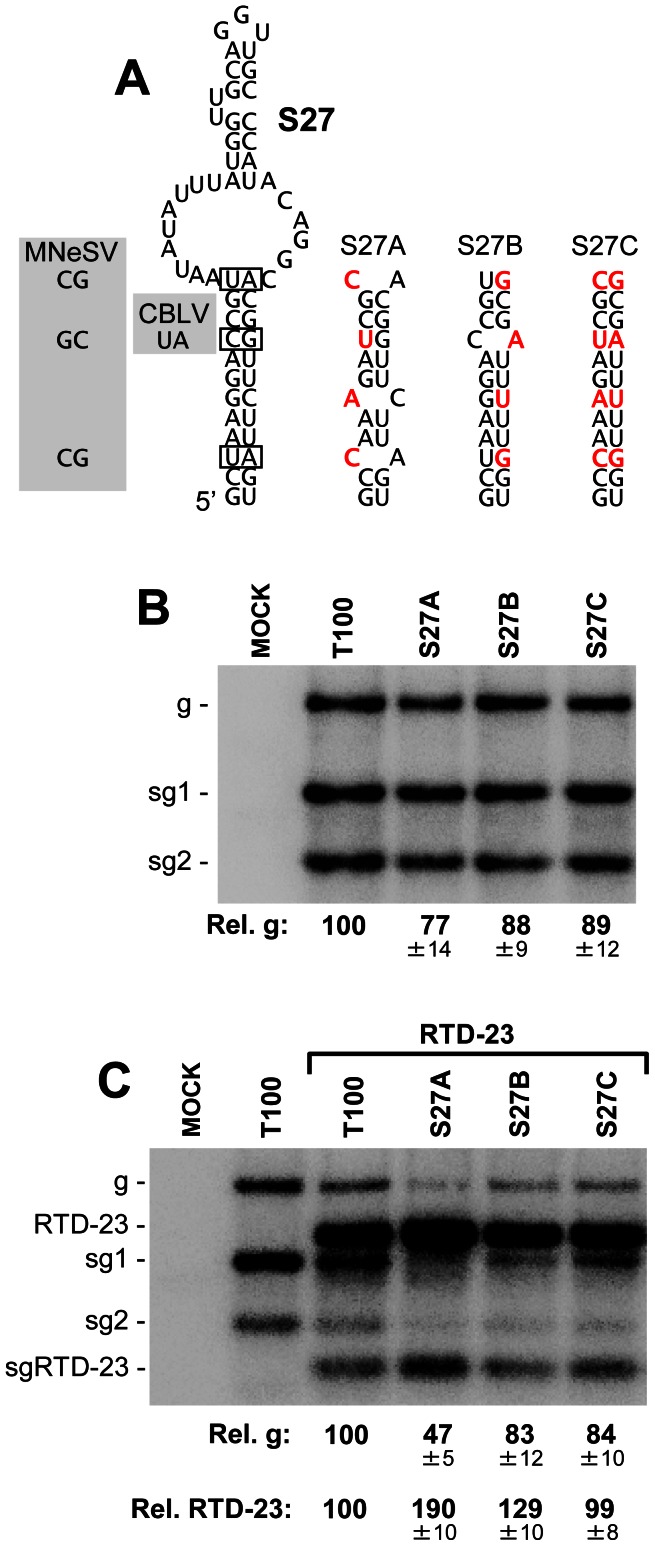
Mutational analysis of the predicted stem-loop 27 structure. (**A**) Predicted structure for stem-loop 27. Base pairs in the structure that covary in MNeSV and CBLV are boxed and the substituted nucleotides are shown in the shaded regions to the left. The compensatory mutants generated are shown to the right with substitutions in red. (**B**) Northern blot analysis of viral RNA accumulation in protoplasts transfected with TBSV genome mutants. The identities of the mutants are indicated at the top of the blot and relative TBSV genome accumulation is shown at the bottom. Values represent means with standard errors from three independent experiments. (**C**) Northern blot analysis of viral RNA accumulation in protoplasts transfected with TBSV genome mutants and replicon RTD-23. Quantification of TBSV genomes and RTD-23 are shown below the blot. Values represent means with standard errors from three independent experiments.

The SHAPE data were incorporated into our analysis with the goal of enhancing the accuracy of the structural model. Assessing the correctness of the entire model from a functional standpoint will be challenging, due to its large size and the likelihood that some of its structural components may not confer strong phenotypes. Indeed, certain regions will undoubtedly play more passive roles and thus be tolerant to structural perturbation, while other sections may be very sensitive to alteration. As an initial test of the model, two RNA structures that were predicted to form by SHAPE-plus analysis, but not by SHAPE-minus analysis, were selected for functional investigation. Accordingly, the results from these analyses would provide some feedback as to the effectiveness of the incorporated SHAPE data. The first structure examined was a local extended stem-loop structure, SL27, within mD1 in the p92 coding region (Figures 3 and 4). This structure is conserved within the genus and base pair covariations were present in two species, maize necrotic streak virus (MNeSV) and cucumber Bulgarian latent virus (CBLV), which maintained the lower stem (Figure 6A). To address the relevance of this structure, compensatory mutational analysis was performed, where the lower stem was destabilized and then restored with nucleotide substitutions. Substitutions were designed so as to maintain the amino acid identity of the encoded p92 ORF and, as mutant S27A contained two less GU base pairs than S27B, the former was predicted to be less stable than the latter (Figure 6A). In S27C, the mutations in S27A and S27B were combined to re-establish canonical base pairing at all substituted positions. When the mutant TBSV genomes containing these modifications were individually transfected into plant protoplasts, only minor differences in accumulation levels were observed (Figure 6B). However, when these genomes were co-transfected with a smaller competitor viral replicon, a TBSV genome with a single internal deletion that removed the CP and p19/22 ORFs, termed RTD-23 [42], clear differences in accumulation were evident (Figure 6C). In particular, the most destabilized mutant, S27A, showed a decrease in accumulation to ∼47%, while the relative level of the RTD-23 replicon increased to ∼190%, when compared to cotransfection with the wt TBSV genome (Figure 6C). In contrast, the S27B and S27C mutants with higher predicted stability showed genome and replicon accumulation profiles more similar to those of the wt TBSV and RTD-23 coinfection (Figure 6C). The observation that S27A with the most destabilized structure was least able to compete effectively against the replicon suggests that this structure confers a fitness advantage at the single cell level and is functionally relevant to the virus.

**Figure 7:**
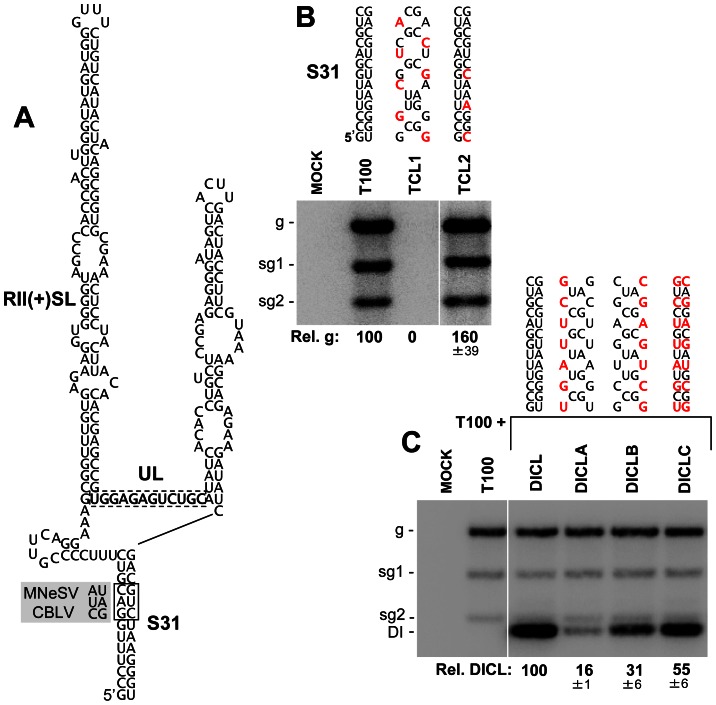
Mutational analysis of the predicted stem-31 structure. (**A**) Predicted structure for S31. Base pairs in the structure that covary in MNeSV and CBLV are boxed by a solid line and the substituted nucleotides are shown in the shaded regions to the left. The UL is boxed by a dotted line and RII(+)SL, which corresponds to S33 in the genome model, is indicated. (**B**) Northern blot analysis of viral RNA accumulation in protoplasts transfected with TBSV genome mutants. The name and changes made in S31 in the genomic mutants are shown above each lane, with substitutions depicted in red. Relative TBSV genome accumulation is shown at the bottom. Values represent means with standard errors from three independent experiments. (**C**) Northern blot analysis of DI RNA accumulation in protoplasts co-transfected with wt TBSV genome and wt or mutant forms of DICL. The changes in S31 in the DICL mutants are indicated for each lane. Quantification of the DI RNAs is shown below the blot. Values represent means with standard errors from three independent experiments.

Next we examined a longer-range 13 base pair long interaction spanning an intervening sequence of 190 nts, which also was predicted only by SHAPE-plus analysis. This interaction formed a helix, S31, that closed mD2 (Figures 3 and 4) and was of particular interest because the intervening sequence included two important RNA replication elements, the large stem-loop structure RII(+)-SL and the UL sequence (Figure 7A). Two species in the genus, MNeSV and CBLV, contained covariations that maintained S31 but, surprisingly, the substitutions in the 5′ half of this stem were not silent and resulted in two amino acid substitutions (Gly_390_Ala and Arg_391_Ser). This finding suggests that, at these positions, maintaining this RNA structure was a higher priority than preserving the consensus amino acid identity in p92. To test the importance of this potential interaction, substitutions were introduced into the complementary sequences of S31 in the TBSV genome. Substitutions were made at degenerate codon positions so as not to change the corresponding amino acids in the p92 ORF. As no directly opposing degenerate codon positions were available, the introduction of silent compensatory mutations was not possible in the genomic context. Instead, two genomic mutants were made, TCL1 and TCL2, which were predicted to either disrupt or stabilize the interaction, respectively (Figure 7B). TCL1 did not accumulate in transfected protoplasts, whereas TCL2 accumulated to greater than wt levels (Figure 7B), consistent with the interaction being functionally relevant.

The location of the interaction close to two RNA replication-related elements (*i.e.* RII(+)-SL and UL) suggested a possible role in mediating genome replication. To address this question in a replication context free from translational constraints, a non-coding defective interfering (DI) RNA replicon containing the interaction was used. The DI RNA, termed DICL, was able to replicate efficiently in co-inoculations with helper T100 (which provided p33 and p92 RNA replication proteins in trans) and, as this replicon was non-coding, it was possible to generate a full set of compensatory mutations within it to test the role of S31 (Figure 7C). DICL-A and DICL-B had disruptions in either half of S31 and accumulated to 16% and 31% the levels of wt DICL (Figure 7C). If these two defects were independent, then combining them would be additive and would lead to a predicted ∼5% level of accumulation. However, when the two mutant halves were united in DICL-C, so as to restore base pairing potential, an accumulation level 55% that of wt was observed (Figure 7C), indicating an important role for S31 formation in the accumulation of the DI RNA. Its requirement in a non-coding replicon suggests that the function of S31 is related to regulating viral genome replication, possibly through modulating the activity of the replication elements in the intervening sequence. The apparent phenotype observed for disruption of S31 contrasts the more subtle differences seen for S27 mutants, however both results help to functionally validate components of the SHAPE-plus model and provide a sampling of the range of phenotypes that can be expected in future structure-function analyses of the model.

### Utility of information on viral genome structure

As mentioned previously, the TBSV genome structure is intimately involved in the fundamental viral processes of protein translation, genome replication, and sg mRNA transcription. Naturally, the structural knowledge gained in this study will be useful to design additional experiments to further explore these basic processes. However, genome structure is also relevant to several other aspects of viral infections. One such area is virus-induced gene silencing (VIGS), an antiviral host defence system that is based on the detection and destruction of viral RNA [44], [45]. Detection is mediated by the enzyme Dicer, which binds to and cleaves double-stranded viral RNA into small interfering RNAs (siRNAs) that are used as guides by the RNA-induced silencing complex (RISC) to target and cleave viral genomes [44], [45]. Possible targets for Dicer include viral double-stranded RNA replication intermediates [46] and highly structured regions of viral RNA genomes [47]–[49]. With respect to the latter possibility, our current structural model predicts several regions with extended uninterrupted helices, which represent possible targets for Dicer. Although in vivo structures may differ somewhat from our model, it represents a good starting point for mapping and correlating Dicer hotspots with structured regions. Our model would also be useful for in vitro studies using plant-derived Dicer extracts [50], [51] along with in vitro transcripts of the viral genome, which would allow for direct assessment of substrate preferences. Additionally, since the efficiency of RISC-based cleavage of a target RNA is known to be inhibited by secondary structure in the target sequence [52], [53], knowing which regions are less structured and, thus, better potential targets, would be beneficial for designing RNAi-based antiviral measures.

One of the driving forces in RNA virus evolution is recombination, in which discontinuous regions of a viral genome(s) are joined together [54]. The process of recombination has been studied in many RNA viruses [54], and in tombusviruses the deletion events leading to the formation of small DI RNAs have been used as a model to understand RNA recombination [55]. These deletions presumably occur when the viral polymerase dissociates from its template along with its nascent strand and then rebinds some distance upstream, where it continues nascent strand extension. In vitro and in vivo studies in tombusviruses have provided evidence that discontinuous copying can occur during genomic minus-strand synthesis [56], [57]. In such cases, the viral genome would act as the template and in our structural model the four regions maintained in a prototypical DI RNA are all positioned relatively close to each other (Figure 4). Local sequences and structures are known to mediate polymerase dissociation and/or rebinding [58], [59], however the proximity of these take-off and landing sites, as determined by the global structure, could also aid in this process. Accordingly, global structure is a likely determinant of intramolecular recombination events leading to the generation of deletions in genomic RNA.

For RNA virus characterization, a common strategy used to define the role of a viral protein is to mutate its corresponding ORF in the infectious clone. However, it is becoming increasingly evident that functional RNA elements also reside within coding regions of different RNA viruses [1], [2]. Thus, the modification of a coding region could also alter an overlapping functional RNA structure, which would greatly complicate the interpretation of results. In such cases, a reliable genomic structural model that includes local and long-range interactions would be useful, as this information could be integrated into the interpretation of data to help avoid erroneous conclusions. Additionally, the ability to manipulate infectious transcripts of RNA virus genomes has also allowed for their development as foreign protein expression vectors [60]. Challenges to this application include low levels of vector replication and/or deletion of foreign inserts, both of which lead to low protein yield. Based on our model for TBSV, we can now start to appreciate how insertion of foreign sequences or replacement of viral sequences could lead to disruption of one or more of the components of the global structure. As a result, structural models such as that for the TBSV genome should prove useful in designing viral vectors that are compatible with the natural organization and function of viral genomes.

### Conclusion and perspective

With respect to the reliability of our SHAPE-guided structure, we feel that (i) the general agreement with the AFM structure, (ii) the presence of known local structures, (iii) the occurrence of some of the known long-range interactions, and (iv) identification of new structures that were functionally validated, all add confidence to the prediction. Nonetheless, the accuracy of many aspects of the structure still remains to be investigated. The current structural model provides a context to begin to understand the organization of local structures and long-range RNA-RNA interactions within the TBSV genome. At a global level, the genome assumes a floret-like structure that includes multiple long-range RNA-RNA interactions. The simultaneous presence of the AS1-RS1 and DE-CE interactions in our model suggests that transcriptional-related interactions are structurally compatible and points to functional coupling of this process. Conversely, the absence of other known long-distance interactions in the structure implies the requirement for dynamic structural transitions, some of which may be mutually exclusive. However, the basic framework of secondary structure observed may mediate the formation of alternate structures without the need for large-scale rearrangements. Importantly, our structure provides a foundation to further test and refine the model and to identify alternative global structures that allow other long-range interactions to occur. Such dynamic transitions may occur readily in a cellular environment that includes both viral and host proteins that could mediate structural rearrangements. A possible candidate for this role is the abundant viral protein p33, which has been shown to possess RNA chaperone activity [61]. Future structural studies will need to investigate different experimental conditions in order to develop a comprehensive understanding of all relevant genomic conformations. From a comparative perspective, as the structures of additional RNA viral genomes are determined, it will be interesting to see if related viruses (*e.g.* different genera in the same family) display any likeness at the global genomic level and if unrelated viruses share similar organizational features that can help to define general rules of arrangement.

## Materials and Methods

### Plasmid construction

The infectious clone of the wild-type TBSV genome, T100 [22], the replicon RTD-23 [42] and TBSV DI RNAs [55] have been described previously. Mutant TBSV genomes were constructed based on T100, where modifications were introduced using PCR-based mutagenesis and standard cloning techniques. The PCR-derived regions in all constructs were sequenced completely to ensure that only the intended modifications were present. The modifications introduced into the mutants are shown in the accompanying figures.

### AFM sample preparation and imaging

For AFM imaging, TBSV RNA genome derived from in vitro transcription of clone T100 was diluted to 1.5 ng/µl in deposition buffer (20 mM HEPES, pH 7.08, 10 mM MgCl_2_). A 20 µl volume of the diluted RNA sample was deposited on freshly cleaved mica and incubated for 1 min at room temperature. Following the incubation period, the sample was rinsed with double distilled water, dried under a stream of nitrogen gas, and imaged using AFM tapping mode in air. Tapping mode AFM was performed with a Dimension 3100 microscope and Nanoscope IIIa controller (Digital Instrument Inc., Vecco). Tapping mode AFM images in air were obtained using a silicon cantilever-tip assembly with a resonance frequency of 200–300 kHz. The images were captured at scan rate of 1–1.5 Hz and 512×512 pixels resolution. Image height scale was typically of the order of ca. 3 nm; exact scales are provided for each image. Images were viewed and analyzed using V614r1 Nanoscope program (Digital Instrument Inc.). Images were subjected to third order flattening to remove image bowing artifacts.

### In vitro transcription, protoplast transfection, and viral RNA analysis

Viral RNAs were generated by in vitro transcription using T7 RNA polymerase as described previously [55]. Protoplasts (∼300,000) derived from cucumber cotyledons were transfected with viral RNA transcripts (3 µg for genomic RNA; 1 µg for replicon or DI RNA) using polyethylene glycol [18] and incubated at 22°C or 28°C for 22 hr. Total nucleic acids were extracted from transfected protoplasts using phenol/chloroform. Following ethanol precipitation they were separated in 1.4% agarose gels and subjected to Northern blot analysis as described previously [18]. Equal loading for all samples was confirmed prior to transfer via staining the gels with ethidium bromide. Viral RNAs were detected using strand-specific ^32^P-labeled probes and relative isotopic levels were determined using PharosFx Plus Molecular Imager.

### RNA preparation for SHAPE Analysis

High-throughput SHAPE analysis was carried out using the methodology described by Low and Weeks [33]. TBSV genomic RNA was prepared by in vitro transcription using AmpliScribe T7-Flash Transcription Kits, and subsequently purified by two rounds of ammonium acetate precipitation. Approximately 70% of the genomic TBSV RNA was determined to be intact, as estimated by ethidium bromide staining after gel electrophoresis. Eighty pmol of TBSV RNA in 480 µL of 0.5× TE buffer (5 mM Tris, pH 8, 0.5 mM EDTA) was heated for 5 min at 95°C and then transferred to ice for 2 min. Subsequently, 240 µL of 3.3× folding buffer (333 mM HEPES pH 8.0, 16.5 mM MgCl_2_, 333 mM NaCl) was added, followed by incubation at 37°C for 30 min.

### RNA modification

Three hundred and sixty µL of the folded RNA was treated with 40 µL of 50 mM 1-methyl-7-nitroisatoic anhydride (1M7) in DMSO and another 360 µL aliquot of the folded RNA was treated with 40 µL neat DMSO. Both were subsequently incubated at 37°C for 4 min. 1M7-modified RNA (+), and DMSO control RNA (−) were recovered by ethanol precipitation (with 200 mM NaCl, 2 mM EDTA, and 800 µg glycogen) and resuspended in 400 µL of 0.5× TE.

### Primer design and synthesis

TBSV genomic RNA secondary structure was predicted by *Mfold* RNA folding software [62] and complementary DNA primers were designed for regions predicted to lack strong RNA secondary structure. In total, 19 primers were designed to cover the TBSV genome (Table S3) with an average coverage distance of ∼360 nt per primer (Table S4). Each primer was separately synthesized with 4 different florescent dyes (WellRED D2, WellRED D3, WellRED D4, and LICOR IR 800) at the 5′end and purified by reverse-phase cartridge purification method (Sigma-Genosys).

### Primer extension

Fluorescently-labeled primer (6 µL at 4 µM) was added to 20 µL RNA (+) (4 µM WellRED D4) and 20 µL RNA (−) (4 µM WellRED D3) reactions and incubated at 65°C for 5 min and 37°C for 5 min. Twelve µL SHAPE enzyme mix (167 mM Tris-HCl, pH 8.3, 250 mM KCl, 10 mM MgCl_2_ 16.7 mM DTT, 1.67 mM dATP, 1.67 mM dTTP, 1.67 mM dCTP, 1.67 mM dITP) was added to each tube and incubated at 52°C for 2 min, to which 1.5 µL of SuperScript III (Invitrogen) was added and incubated at 52°C for 30 min. The extension reaction was stopped by incubating on ice for 2 min and then adding 4 µL of 3 M NaOAc (pH 5.2). For sequencing reactions, 7.2 pmol RNA in 40 µL of 0.5× TE was incubated at 95°C for 4 min and transferred to ice for 2 min to denature the RNA. Six µL primer (4 µM, WellRED D2) was added to 20 µL denatured RNA (Seq-G) and 6 µL primer (4 µM, LICOR IR 800) was added to another aliquot of 20 µL denatured RNA (Seq-T). After primer and RNA annealing at 65°C for 5 min and then 37°C for 5 min, 2 µL of ddGTP (0.5 mM) and 2 µL ddTTP (10 mM) were added to Seq-G and Seq-T, respectively, and the rest of the procedure was as described above for the 1M7-treated and untreated samples. The four reactions were combined and precipitated with ethanol and 2 µL of glycogen (20 mg/ml). The cDNA pellet was washed twice with 70% ethanol, dried, and resuspended in 40 µL of deionized formamide.

### Capillary electrophoresis

cDNA samples were separated in a 33 cm long (75 µm inner diameter) capillary using a Beckman CEQ800 DNA sequencer. Samples were denatured at 90°C for 120 seconds, injected at 2.0 kV for 6 to 15 sec, and separated at 4 kV for 80 min.

### Data processing

SHAPE electropherogram intensities were quantified using *SHAPEfinder* [63]. Briefly, 1) The hSHAPE data, as esd files, were loaded from the capillary electrophoresis instrument. 2) Using the base line adjustment tool, drifting baselines in each channel were corrected, with a window width of 10 times the inter-peak distance. All the channels had a common baseline. 3) The matrixing tool was used for color separation to make sure that each channel represents one dye amount, and not fluorescent intensity from the other three dyes. 4) The differences in electrophoretic migration rate of the cDNA products caused by different fluorophores were corrected using cubic mobility shift tool. 5) Because of the imperfect processivity of the reverse transcriptase, signals decay from left to right in hSHAPE electropherograms were corrected using the signal decay correction tool. Normally, the entire area of interpretable data was selected for the correction. 6) The scale of each channel in a trace was adjusted using the scale factor tool. For accurate subtraction of background intensities, corresponding to the small peak positions in the (+) channel, the peak sizes in the (−) channel were adjusted to the same intensities as the peaks in the (+) channel. 7) The align and integrate tool was used to calculate and align nucleotide activity to the RNA sequence. There are three phases in this tool. First, in the “setup” phase, every SHAPE reaction was assigned to a channel; the sequencing peak sensitivity was adjusted; the data range to be analyzed was specified; the RNA sequence file corresponding to the hSHAPE experiment was loaded. Second, in “modify” phase, the sequence offset created in the “setup” phase by missing and adding a peak was corrected by adding and deleting a peak. Finally, in the “fit” phase, the reactivity of each nucleotide was calculated. Reactivities were assigned to 4347 nts (∼91% of the genome). Due to blocks in reverse transcription, SHAPE information for the contiguous regions 1008–1163 nts and 2464–2629 nts could not be collected. Quality of SHAPE data of 9 nts at the 5′ end and 83 nts at 3′ end was not collected due to the low quality data at 5′ end and the absence of data at the 3′end. Fifteen nts throughout the entire genome had low quality data (e.g. peaks in 4 channels at one nucleotide position were high). Accordingly, SHAPE data for these regions or nts (colored in grey, Figure 3; Table S1) were not included in the computational folding (*i.e.* structural prediction in these regions was based only on thermodynamics.) For the rest of the genome, the raw SHAPE reactivities were normalized using the box-plot normalization method [63] in these steps: 1) All data lying outside 1.5 times the interquartile range were identified as outliers; 2) the normalization factor was calculated as the average of the top 10% in the remaining data; and 3) all data (including outliers) were then multiplied by the reverse of the normalization factor. 4) These data were smoothed by using the sigmoid function
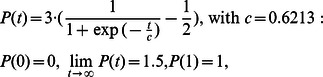

*i.e.* the normalized reactivity was set to  = 1 if t was equal to the average of the top 10% non-outlier data. These preprocessing steps were carried out in MATLAB.

### Computational structure prediction

#### SHAPE-plus structure prediction

The average reactivity at each position from two separate SHAPE analyses of the TBSV genomic RNA were incorporated into the *RNAstructure* software [33], [64] choosing pseudo-free energy parameters of m = 2.6 kcal/mol (slope) and b = −0.8 kcal/mol (intercept). Formation of the confirmed local RNA structures in the 5′UTR (nts 1–166 ) and 3′UTR (nts 4697–4778) was added as a constraint in the input file for *RNAstructure*. No limit was imposed on the maximal base pairing range (which is the default in *RNAstructure*). Genome RNA structures were generated using *RnaVis2* software [65]. Varying the pseudo-free energy parameters within 0.1 of the default values yielded structures with domain organizations that were consistent with that of the structure generated with the default values (Figure S5) and none of these structures predicted the 5′UTR-3′CITE, UL-DL, PRTE-DRTE, or AS2-RS2 interactions (Figure S6).

Suboptimal structures: 1000 structures each were stochastically sampled from the ensemble by using i) the SHAPE-constrained .pfs file and ii) the thermodynamic pfs file generated in *RNAstructure*, and by using the constraints at the 5′ and 3′ ends. The base-pairing probability pfs file, which is a prerequisite in *RNAstructure* for drawing a stochastic sample from the ensemble, could not be successfully generated in the software by simultaneously placing restraints (i.e. SHAPE data) and hard constraints (at the 5′ end 3′ ends, i.e. those based on previous experimental results). To circumvent this limitation we followed a modified procedure in simulating a sample of plus-SHAPE suboptimal structures: For the SHAPE-constrained structures the pfs file was generated over the range of nts 167–4696 (i.e. excluding the terminal regions that were constrained and corresponding to ∼98% of the genome), and the 1000 sampled structure were concatenated with the separate foldings of 5′ and 3′ ends (nts 1–166, nts 4697–4778, resp.), for which latter the constraints, but no SHAPE reactivity data restraints, were applied. Suboptimal structures were processed and interrogated for alternative or additional interactions not seen in the SHAPE-plus mfe structure, using MATLAB scripts.

Mountain plots and maximum ladder distance calculations: Mountain plots of the SHAPE-plus structure, suboptimal structures, and structures of 8 incremental variations in pseudo-free energy parameters (m,b) = (2.6,−0.8)+/−(0.1, 0.1) were generated with MATLAB scripts.

The maximum ladder distance (MLD) for the SHAPE-plus and SHAPE-minus structures and their mean values <MLD> over the ensembles values for i) 1000 SHAPE-plus sampled structures and ii) 1000 SHAPE-minus sampled structures were calculated together with their maximum and minimum values over the ensembles.

Computational analyses were carried out via Perl/Python and MATLAB scripts interfacing with the *RNAstructure* software [33] on a Dell PowerEdge R710 bioinformatic server with 2 Intel Xeon X5660 2.80 GHz processors and 48 GB memory.

## Supporting Information

Figure S1 **Accumulation of TBSV genome in transfected plant protoplasts.** Cucumber protoplasts were transfected with 1 or 3 ug of viral RNA transcripts that either were or were not subjected to the RNA folding protocol as describe In the Material and Methods section. Total nucleic acids were extracted at 22 hr post-transfection, separated in a 1.4% agarose gel, and stained with ethidium bromide. The position of the accumulated TBSV viral genomes is indicated.
(TIF)

Figure S2 **Mountain plots for SHAPE-plus TBSV genome optimal and suboptimal structures.** The red graph line indicates the mountain plot for the optimal structure. The graph lines in black above and below the red graph line show, respectively, the maximum and minimum values for the number of enclosed basepairs along the sequence in the sampled 1000 suboptimal structures. Below, the corresponding assigned domains are indicated. The maximum and minimum enclosure values within the suboptimal population mirror that for the optimal structure, indicating that global structure is largely maintained and the domain boundaries are well preserved within the suboptimal population.
(TIF)

Figure S3 **Dot plots for SHAPE-plus TBSV genome optimal and suboptimal structures.** Dot plot showing interactions for the optimal (red dots) and 1000 sampled suboptimal structures (black dots), with annotation of the general areas in which the known 6 long-range interactions in the TBSV genome would reside (blue boxes). The four interactions absent in the optimal structure (*i.e.* 5′UTR-3′CITE, PRTE-DRTE, UL-DL and AS2-RS2) are also absent in all suboptimal structures.
(TIF)

Figure S4 ***RNAstructure*** **-predicted secondary structure for the TBSV genome.** No SHAPE data were used in this prediction. Formation of the confirmed local RNA structures in the 5′UTR (nts 1–166) and 3′UTR (nts 4697–4778) were added as constraints in the input file.
(TIF)

Figure S5 **Mountain plots of SHAPE-plus structures generated with varying pseudo-free energy parameters.** The pair-wise values used in variants were: m = 2.5, 2.6, 2.7; b = −0.7, −0.8, −0.9. The structure for the default parameter (*i.e.* m = 2.6, B = −0.8) is shown in red, the maximum and minimum values for number of enclosed basepairs along the sequence in the sampled parameter variants are shown in blue. Good robustness of overall domain structure with respect to incremental changes in (m,b) was seen in the simulations. In each pairwise comparison, more than 90% of the basepairs occurring in the structure with the default parameters were identical to the basepairs in the structures with parameter variants. We were not able to compute stable structures for larger parameter variations, due to software instability for large RNAs with enforced constraints. Below the graph the corresponding assigned domains are indicated.
(TIF)

Figure S6 **Dot plots for SHAPE-plus TBSV genome structures.** Dot plot showing interactions for the SHAPE-plus optimal structure generated using the default values (m,b) = (2.6, −0.8) (red dots) and those in 8 SHAPE-plus optimal structures using parameter variations (m,b) = (2.6, −0.8)+/−(0.1, 0.1) (black dots). The general areas in which the known 6 long-range interactions in the TBSV genome would reside are denoted by blue boxes. The four interactions absent in the optimal structure (i.e. 5′UTR-3′CITE, PRTE-DRTE, UL-DL and AS2-RS2) are also absent in all 8 variant structures.
(TIF)

Table S1 **Shape reactivities for the TBSV genome.**
(XLSX)

Table S2 **Shape reactivities for complementary partner RNA elements that did not form cognate pairs in the SHAPE-plus structure.**
(DOC)

Table S3 **List of primers used for SHAPE analysis along with their coordinates relative to the TBSV genome sequence.**
(DOC)

Table S4 **Primer coverage and overlap in primer extension analysis.** For each primer, the nucleotides for which reactivity data was generated is shown under “Covered nt”, while the number of nucleotides of overlap with that from the next primer is shown under “# of overlap”.
(DOC)
