# Supplementary figures and images for: Global Organization of a Positive-strand RNA Virus Genome
Source: PLoS Pathog. 2013 May 23;9(5):e1003363. doi: 10.1371/journal.ppat.1003363 (PMC3662671; doi:10.1371/journal.ppat.1003363)

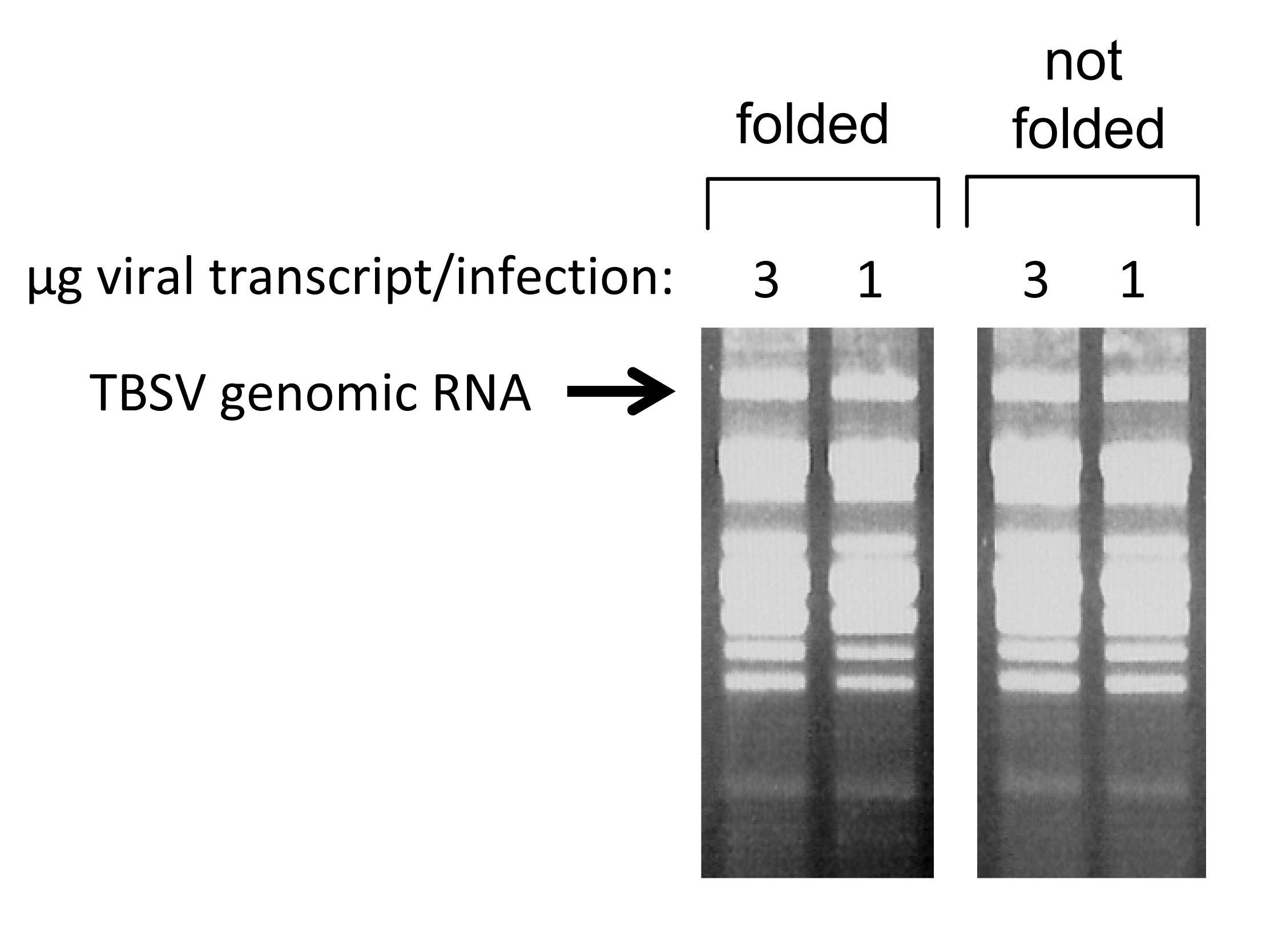

Supplement: Figure S1 — Accumulation of TBSV genome in transfected plant protoplasts. Cucumber protoplasts were transfected with 1 or 3 ug of viral RNA transcripts that either were or were not subjected to the RNA folding protocol as describe In the Material and Methods section. Total nucleic acids were extracted at 22 hr post-transfection, separated in a 1.4% agarose gel, and stained with ethidium bromide. The position of the accumulated TBSV viral genomes is indicated. (TIF) [file ppat.1003363.s001.tif]

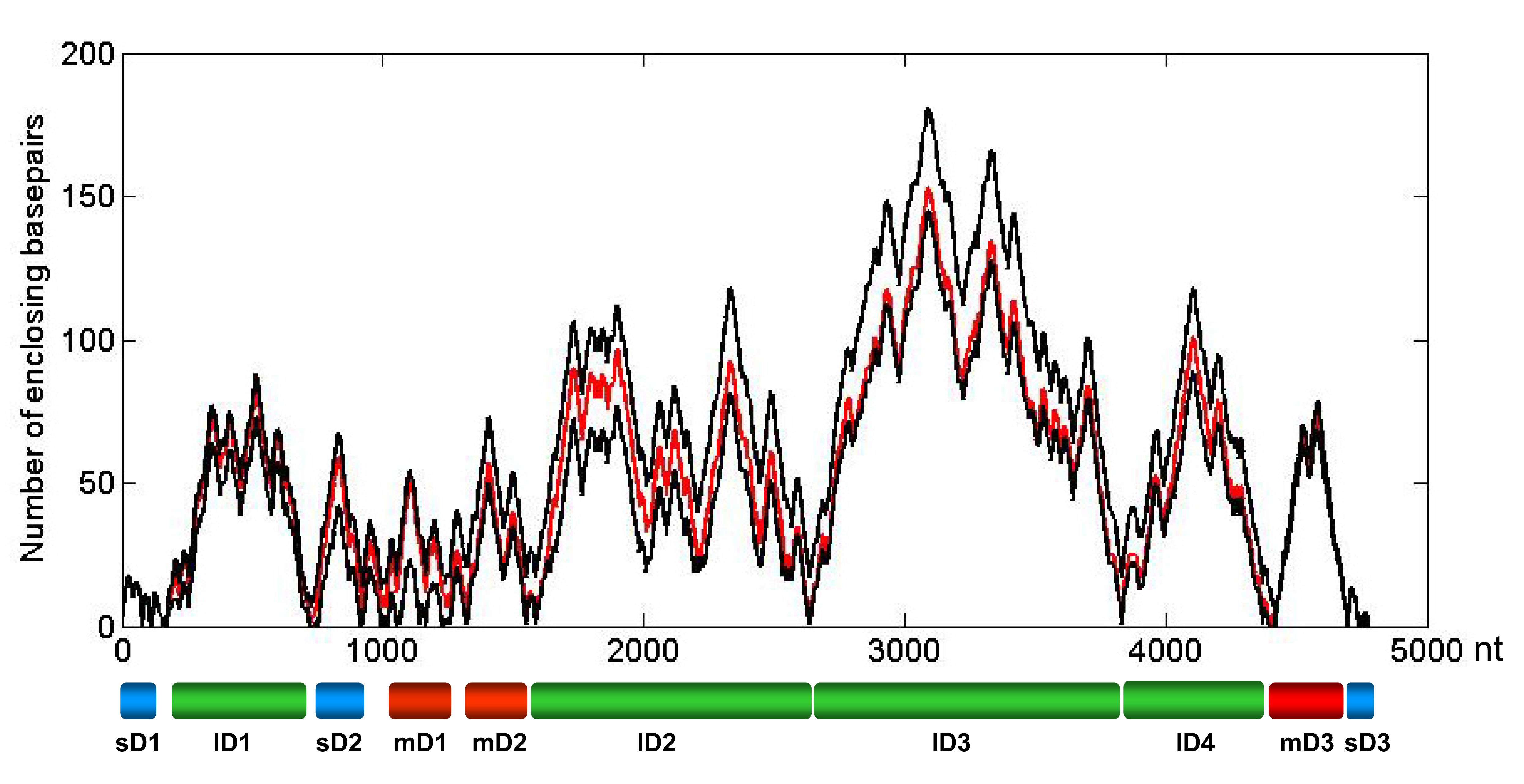

Supplement: Figure S2 — Mountain plots for SHAPE-plus TBSV genome optimal and suboptimal structures. The red graph line indicates the mountain plot for the optimal structure. The graph lines in black above and below the red graph line show, respectively, the maximum and minimum values for the number of enclosed basepairs along the sequence in the sampled 1000 suboptimal structures. Below, the corresponding assigned domains are indicated. The maximum and minimum enclosure values within the suboptimal population mirror that for the optimal structure, indicating that global structure is largely maintained and the domain boundaries are well preserved within the suboptimal population. (TIF) [file ppat.1003363.s002.tif]

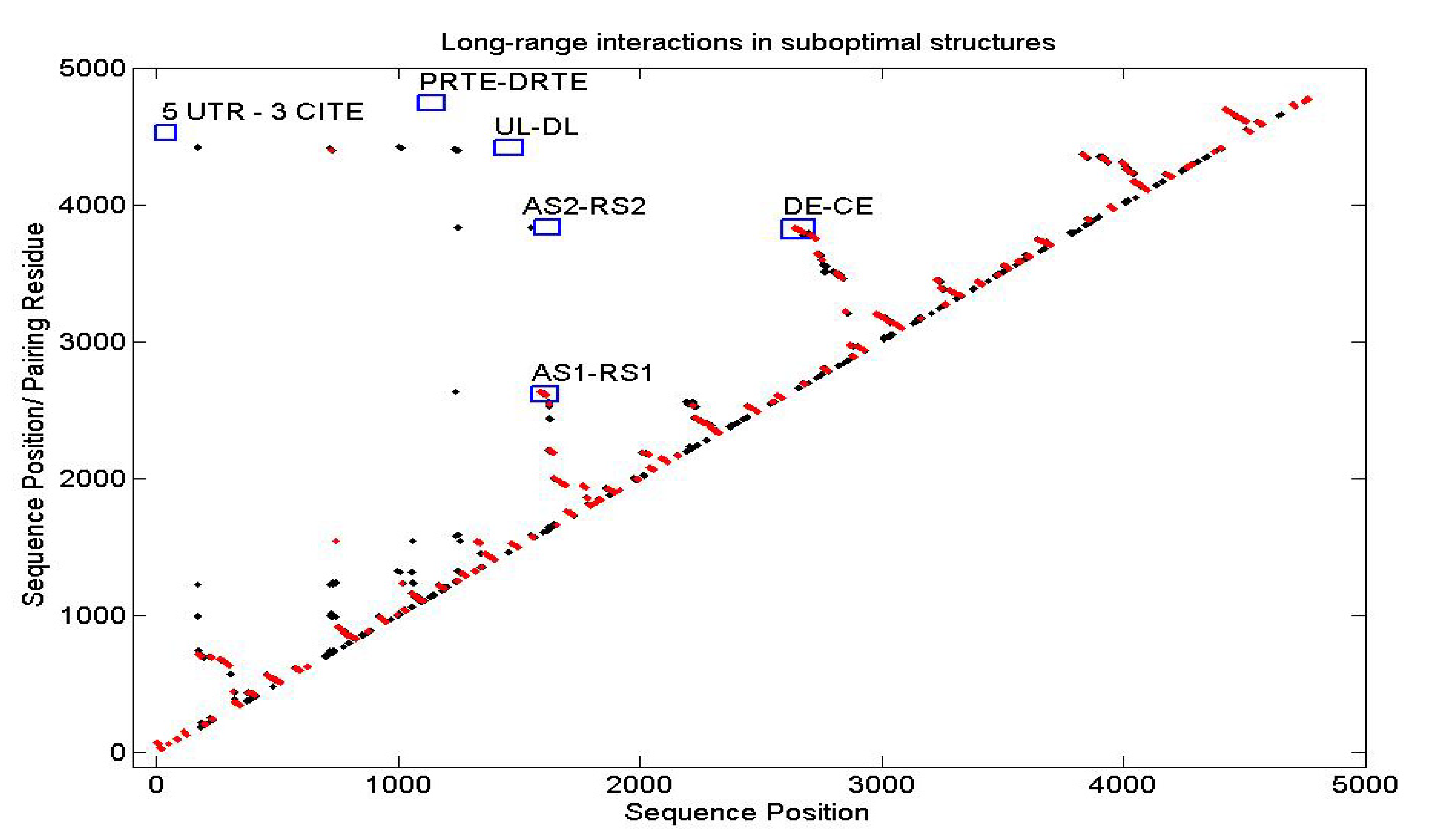

Supplement: Figure S3 — Dot plots for SHAPE-plus TBSV genome optimal and suboptimal structures. Dot plot showing interactions for the optimal (red dots) and 1000 sampled suboptimal structures (black dots), with annotation of the general areas in which the known 6 long-range interactions in the TBSV genome would reside (blue boxes). The four interactions absent in the optimal structure (i.e. 5′UTR-3′CITE, PRTE-DRTE, UL-DL and AS2-RS2) are also absent in all suboptimal structures. (TIF) [file ppat.1003363.s003.tif]

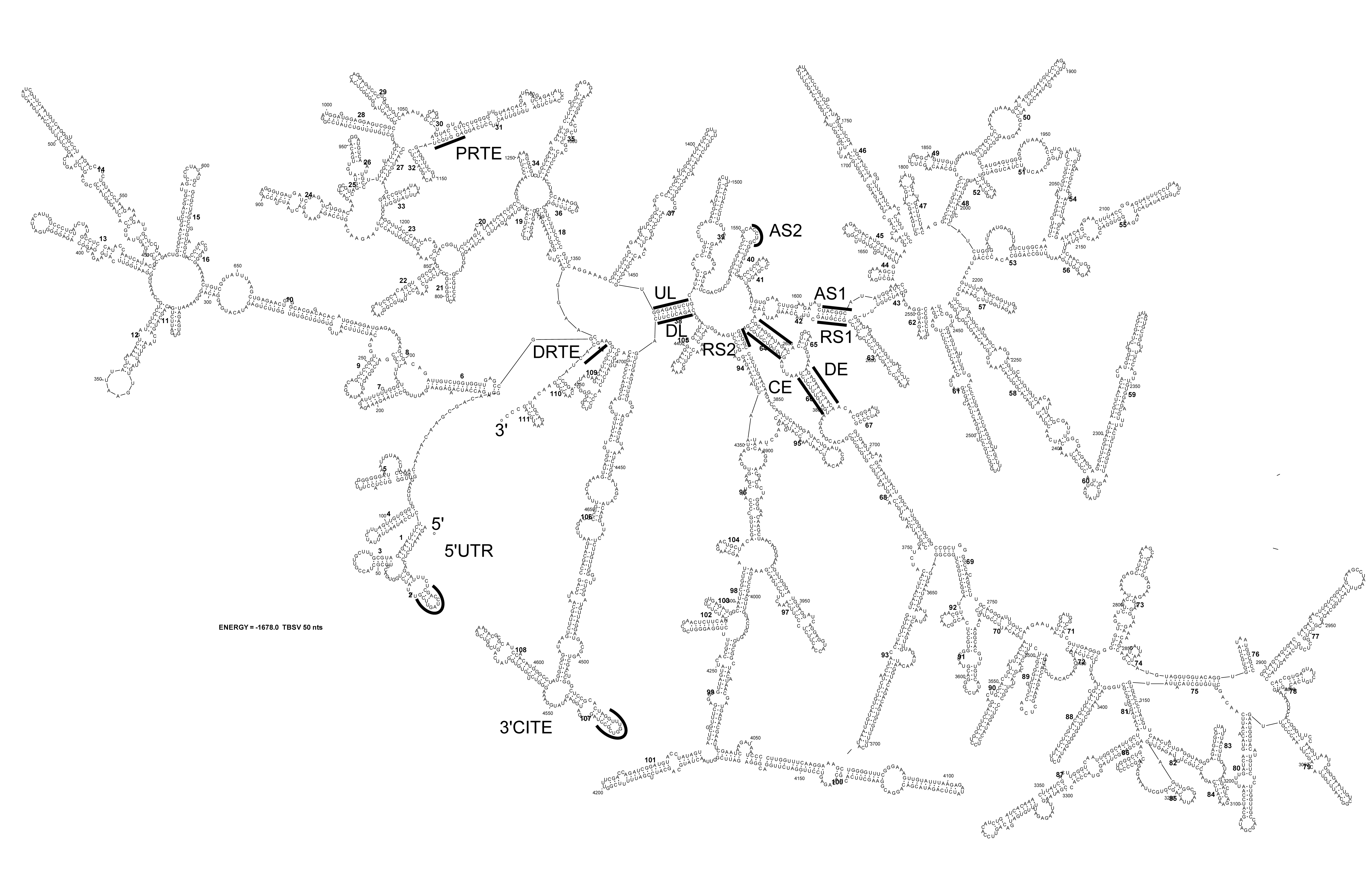

Supplement: Figure S4 — RNAstructure -predicted secondary structure for the TBSV genome. No SHAPE data were used in this prediction. Formation of the confirmed local RNA structures in the 5′UTR (nts 1–166) and 3′UTR (nts 4697–4778) were added as constraints in the input file. (TIF) [file ppat.1003363.s004.tif]

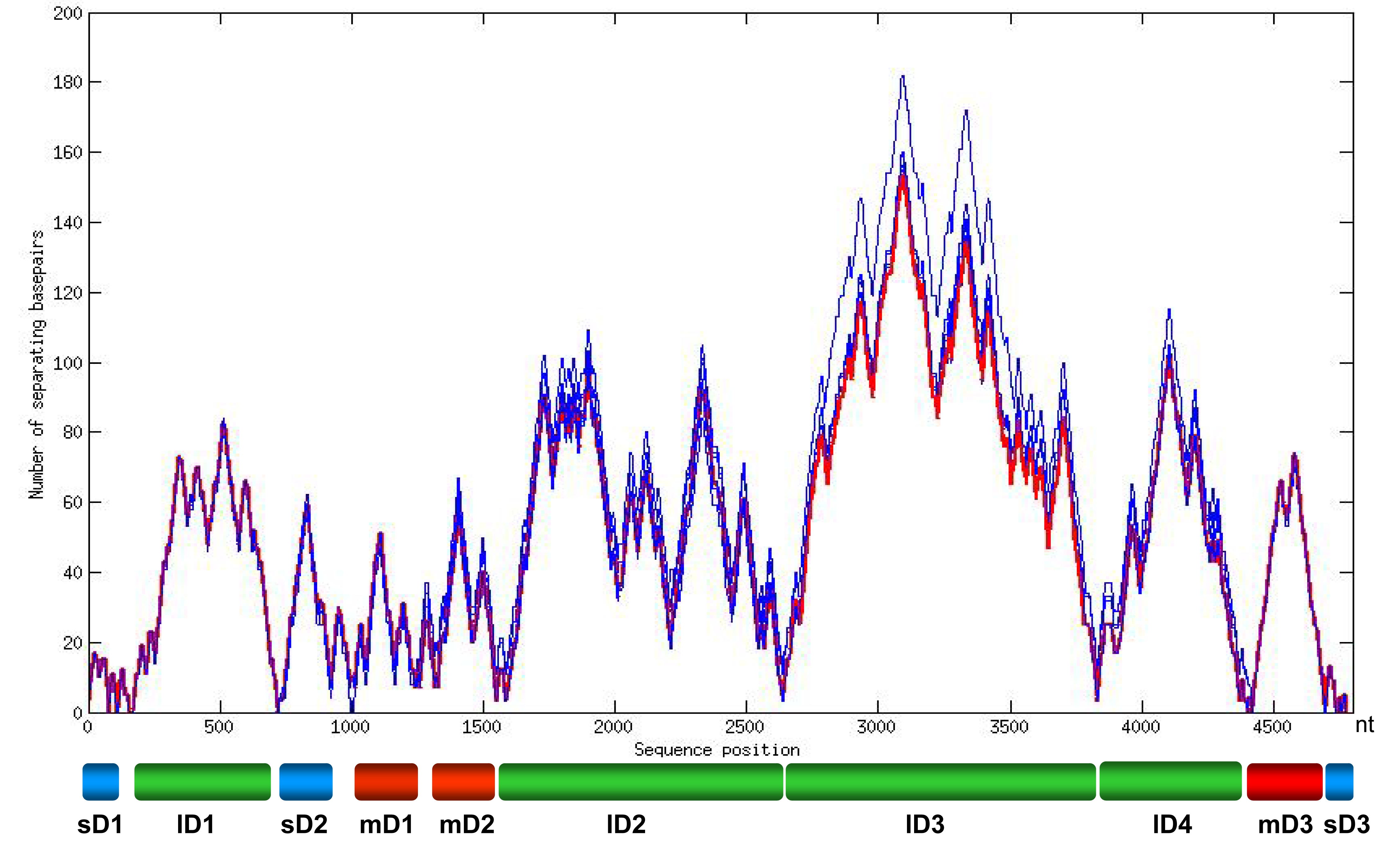

Supplement: Figure S5 — Mountain plots of SHAPE-plus structures generated with varying pseudo-free energy parameters. The pair-wise values used in variants were: m = 2.5, 2.6, 2.7; b = −0.7, −0.8, −0.9. The structure for the default parameter (i.e. m = 2.6, B = −0.8) is shown in red, the maximum and minimum values for number of enclosed basepairs along the sequence in the sampled parameter variants are shown in blue. Good robustness of overall domain structure with respect to incremental changes in (m,b) was seen in the simulations. In each pairwise comparison, more than 90% of the basepairs occurring in the structure with the default parameters were identical to the basepairs in the structures with parameter variants. We were not able to compute stable structures for larger parameter variations, due to software instability for large RNAs with enforced constraints. Below the graph the corresponding assigned domains are indicated. (TIF) [file ppat.1003363.s005.tif]

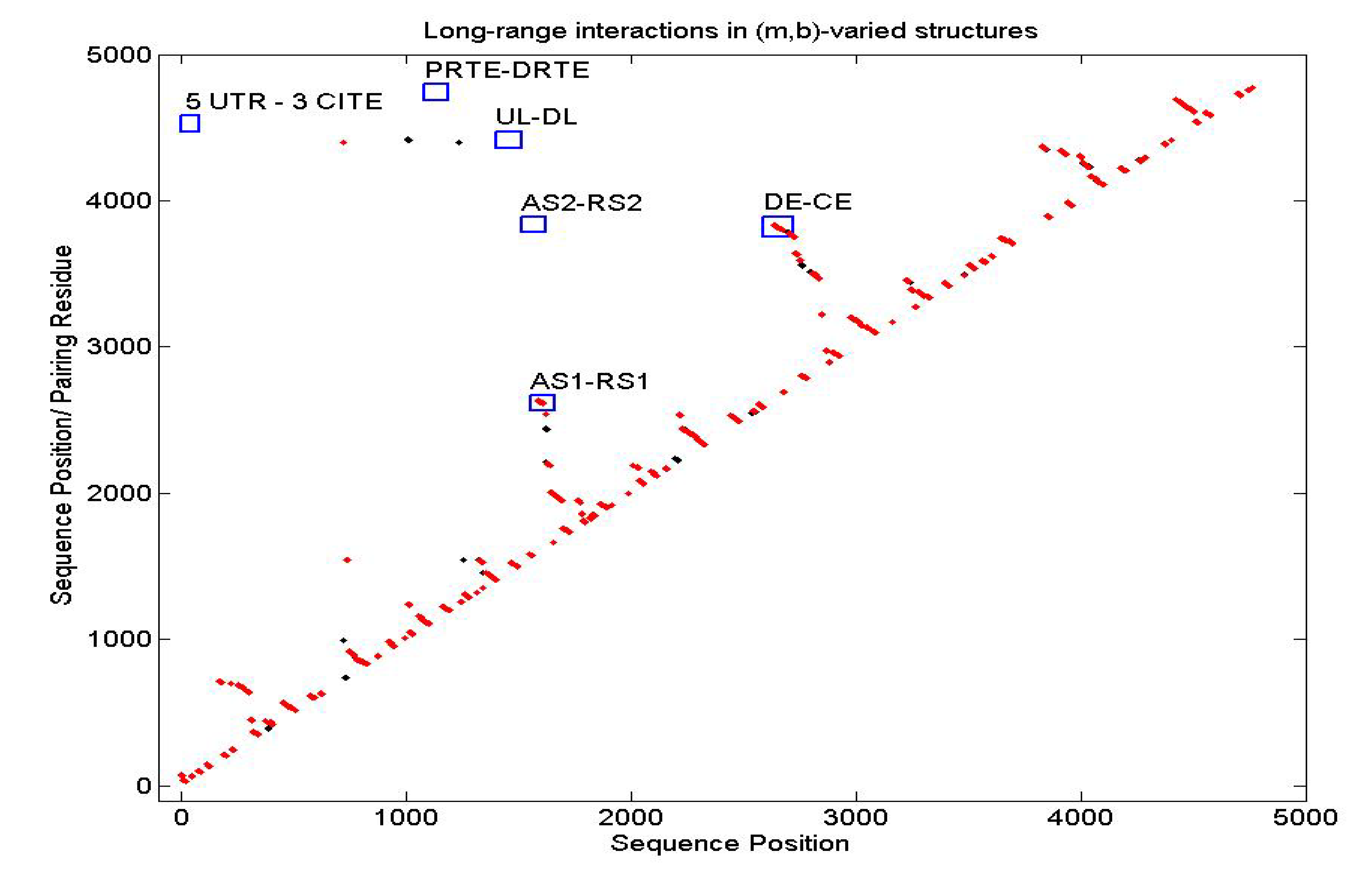

Supplement: Figure S6 — Dot plots for SHAPE-plus TBSV genome structures. Dot plot showing interactions for the SHAPE-plus optimal structure generated using the default values (m,b) = (2.6, −0.8) (red dots) and those in 8 SHAPE-plus optimal structures using parameter variations (m,b) = (2.6, −0.8)+/−(0.1, 0.1) (black dots). The general areas in which the known 6 long-range interactions in the TBSV genome would reside are denoted by blue boxes. The four interactions absent in the optimal structure (i.e. 5′UTR-3′CITE, PRTE-DRTE, UL-DL and AS2-RS2) are also absent in all 8 variant structures. (TIF) [file ppat.1003363.s006.tif]
