# Supplementary material for: Global Organization of a Positive-strand RNA Virus Genome
Source: PLoS Pathog. 2013 May 23;9(5):e1003363. doi: 10.1371/journal.ppat.1003363 (PMC3662671; doi:10.1371/journal.ppat.1003363)
Supplement: Table S2 — Shape reactivities for complementary partner RNA elements that did not form cognate pairs in the SHAPE-plus structure. (DOC) [file ppat.1003363.s008.doc]

**Table S2**: Shape reactivities for complementary partner RNA elements that did not form cognate pairs in the SHAPE-plus structure.

| **Element** | **#*** | **nt** | **SHAPE** |  | **Element** | **#** | **nt** | **SHAPE** |
| --- | --- | --- | --- | --- | --- | --- | --- | --- |
| **5’UTR:** | 21  22  23  24  25  26  27  28  29  30 | C  G  A  C  C  U  A  G  U  U | 0.40889  0.59393  0.262  0.14581  0.70255  1.4333  1.4015  0.13234  1.3592  0.24839 |  | **3'CITE:** | 4531  4530  4529  4528  4527  4526  4525  4524  4523  4522 | G  C  U  G  G  U  U  U  G  G | 0.036065  1.2036  1.3431  1.427  1.2334  1.4994  1.4986  0.05836  0.054542  0.026462 |
| **UL:** | 1455  1456  1457  1458  1459  1460  1461  1462  1463  1464  1465 | U  G  A  G  G  A  G  U  C  U  G | 0.89113  0.30589  0.61645  0.73884  0.76469  0.71215  0.99619  1.1588  1.2035  1.2694  0.54314 |  | **DL:** | 4420  4419  4418  4417  4416  4415  4414  4413  4412  4411  4410 | A  C  U  U  C  U  C  A  G  A  C | 1.4873  0.50069  0.46937  0.24855  0.40661  0.24781  0.25286  0.32838  0.36968  0.51702  0.41309 |
| **AS2:** | 1567  1568  1569  1570  1571 | G  C  C  U  C | 1.1457  0.83017  0.56996  1.1116  1.0218 |  | **RS2:** | 3836  3835  3834  3833  3832 | C  G  G  G  G | 0.24271  0.062794  0.16221  0.17502  0.16994 |

***#**, nucleotide coordinate; **nt**, nucleotide identity; **SHAPE**, reactivity value. Sequences are numbered consecutively and complementary sequences (*i.e.* 5’UTR-3’CITE, UL-DL and AS2-RS2) are oriented antiparallel to each other. Shape reactivity values are color-coded is as in Figure 3.
