# Supplementary material for: Global Organization of a Positive-strand RNA Virus Genome
Source: PLoS Pathog. 2013 May 23;9(5):e1003363. doi: 10.1371/journal.ppat.1003363 (PMC3662671; doi:10.1371/journal.ppat.1003363)
Supplement: Table S3 — List of primers used for SHAPE analysis along with their coordinates relative to the TBSV genome sequence. (DOC) [file ppat.1003363.s009.doc]

**Table S2: List of primers used for SHAPE analysis along with their coordinates relative to the TBSV genome sequence.**

| **Name** | **Position** | **Sequence** |
| --- | --- | --- |
| p9 | (4755-4777) | 5’GGGCTGCATTTCTGCAATGTTCC |
| pTB1 | (4231-4250) | 5’ATACTAACTCCTCGAAACCG |
| pTB2 | (4457-4476) | 5’AACAAGAGTAACCTGTATGC |
| pTB3R | (4005-4024) | 5’TATGTAGCCGCCACTCAGTC |
| pTB4R | (3746-3768) | 5’GGCAGATGTGATACCGGTAGATG |
| pTB5 | (3498-3517) | 5’CAGATGCTGTGACAAGAGCG |
| pTB6 | (3240-3259) | 5’CTTTAAGCACGCTGTAATTC |
| pTB7 | (3025-3044) | 5’AAATTGCCGACAATTCCCCC |
| pTB8 | (2789-2808) | 5’TTTAGTCAGATCGACAGCCC |
| pTB9 | (2542-2561) | 5’GATCCATCTCCCATCTTTCC |
| pTB10R | (2266-2285) | 5’TCGCCAAATCGCGTATATTG |
| pTB11 | (2078-2099) | 5’AAGTTCTCTGTATCTGCTTAAG |
| pTB12 | (1819-1838) | 5’TGTTGCCAGGGTACATGGCC |
| pTB13 | (1591-1610) | 5’GTAGATATCTTCCAAGTTCC |
| pTB14R | (1337-1357) | 5’TTTCCTGAACGGGGAAAGACG |
| pTB15 | (1089-1109) | 5’GGATATCTCGATTGATCTGTG |
| pTB16 | (876-896) | 5’AGATTAGTCTATTCTCTGGAC |
| pTB17 | (633-653) | 5’TAAATACCGACAGTTTTTCCC |
| pTB18 | (372-391) | 5’CATGAACCATTCCCATTTGG |
