# Supplementary material for: Global Organization of a Positive-strand RNA Virus Genome
Source: PLoS Pathog. 2013 May 23;9(5):e1003363. doi: 10.1371/journal.ppat.1003363 (PMC3662671; doi:10.1371/journal.ppat.1003363)
Supplement: Table S4 — Primer coverage and overlap in primer extension analysis. For each primer, the nucleotides for which reactivity data was generated is shown under “Covered nt”, while the number of nucleotides of overlap with that from the next primer is shown under “# of overlap”. (DOC) [file ppat.1003363.s010.doc]

**Table S3: Primer coverage and overlap in primer extension analysis.**  For each primer, the nucleotides for which reactivity data was generated is shown under “Covered nt”, while the number of nucleotides of overlap with that from the next primer is shown under “# of overlap”.

|  | **Repeat-1** | | **Repeat-2** | |
| --- | --- | --- | --- | --- |
| **Primer** | **Covered nt** | **# of overlap** | **Covered nt** | **# of overlap** |
| pTB18 | 10-285 | 34 | 11-296 | 66 |
| pTB17 | 252-570 | 75 | 231-543 | 96 |
| pTB16 | 497-788 | 116 | 448-796 | 109 |
| pTB15 | 673-1007 |  | 688-990 |  |
| pTB14R |  |  |  |  |
| pTB13 | 1164-1519 | 102 | 1209-1521 | 120 |
| pTB12 | 1418-1702 | 85 | 1402-1655 | 9 |
| pTB11 | 1618-1975 | 174 | 1647-2012 | 153 |
| pTB10R | 1802-2163 | 185 | 1860-2158 | 86 |
| pTB9 | 1979-2462 |  | 2073-2463 |  |
| pTB8 |  |  |  |  |
| pTB7 | 2647-2936 | 140 | 2630-2890 | 32 |
| pTB6 | 2797-3163 | 26 | 2859-3178 | 118 |
| pTB5 | 3138-3426 | 111 | 3061-3405 | 129 |
| pTB4R | 3316-3679 | 95 | 3277-3668 | 76 |
| pTB3R | 3585-3900 | 130 | 3593-3917 | 158 |
| pTB1 | 3771-4158 | 112 | 3760-4163 | 168 |
| pTB2 | 4047-4380 | 34 | 3996-4374 | 48 |
| P9 | 4347-4695 |  | 4327-4671 |  |
